# Structure and composition of microbial communities in the water column from Southern Gulf of Mexico and detection of putative hydrocarbon‐degrading microorganisms

**DOI:** 10.1111/1758-2229.13264

**Published:** 2024-05-01

**Authors:** Sonia S. Valencia‐Agami, Daniel Cerqueda‐García, Abril M. Gamboa‐Muñoz, M. Leopoldina Aguirre‐Macedo, José Q. García‐Maldonado

**Affiliations:** ^1^ Instituto de Ciencias del Mar y Limnología, Universidad Nacional Autónoma de México Mexico City Mexico; ^2^ Centro de Investigación y de Estudios Avanzados del Instituto Politécnico Nacional, Unidad Mérida, Departamento de Recursos del Mar Merida Yucatán Mexico; ^3^ Clúster Científico y Tecnológico BioMimic®, Red de Manejo Biorracional de Plagas y Vectores Instituto de Ecología, AC–INECOL Xalapa Veracruz Mexico

## Abstract

This study assessed the bacterioplankton community and its relationship with environmental variables, including total petroleum hydrocarbon (TPH) concentration, in the Yucatan shelf area of the Southern Gulf of Mexico. Beta diversity analyses based on 16S rRNA sequences indicated variations in the bacterioplankton community structure among sampling sites. PERMANOVA indicated that these variations could be mainly related to changes in depth (5 to 180 m), dissolved oxygen concentration (2.06 to 5.93 mg L^−1^), and chlorophyll‐a concentration (0.184 to 7.65 mg m^3^). Moreover, SIMPER and one‐way ANOVA analyses showed that the shifts in the relative abundances of *Synechococcus* and *Prochlorococcus* were related to changes in microbial community composition and chlorophyll‐a values. Despite the low TPH content measured in the studied sites (0.01 to 0.86 μL L^−1^), putative hydrocarbon‐degrading bacteria such as *Alteromonas*, *Acinetobacter*, *Balneola*, *Erythrobacter*, *Oleibacter*, *Roseibacillus*, and the MWH‐UniP1 aquatic group were detected. The relatively high copy number of the *alkB* gene detected in the water column by qPCR and the enrichment of hydrocarbon‐degrading bacteria obtained during lab crude oil tests exhibited the potential of bacterioplankton communities from the Yucatan shelf to respond to potential hydrocarbon impacts in this important area of the Gulf Mexico.

## Graphical abstract

This study analysed the bacterioplankton communities from the Yucatan shelf which could potentially respond to latent hydrocarbon impacts in this significant area of the Gulf of Mexico. Based on changes in relative abundances and increases in *alkB* gene copies, this work suggests that Alteromonas could be considered a keystone microbial group for hydrocarbon degradation in the studied area.

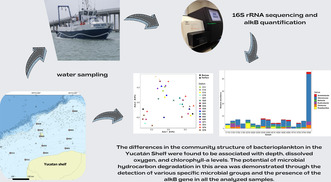

## INTRODUCTION

Planktonic bacterial and archaeal communities (bacterioplankton) are the main controllers of ocean biogeochemical cycles (Bunse & Pinhassi, 2017; Richa et al., 2017; Tsementzi et al., 2016; Wang et al., 2017). Temperature, salinity, nutrients availability and organic matter, among other physicochemical parameters (particular to each water body), are closely related to the composition of the microbial communities associated with the water column (Bastida et al., 2016; Carlson & Hubert, 2019; Fontes et al., 2018; Fortunato et al., 2012; Gilbert et al., 2012; Giovannoni & Vergin, 2012; Hewson, Steele, et al., 2006; Lindh & Pinhassi, 2018; Morris et al., 2018). Consequently, variations in physicochemical conditions can influence the diversity and richness of microbial communities, as well as the functionality of ecosystems (Fortunato et al., 2012; Giovannoni & Vergin, 2012; Hewson, Steele, et al., 2006; Tsementzi et al., 2016). Current studies have shown that physicochemical variations associated with anthropogenic disturbances can impact ecosystem functionality (Bastida et al., 2016; Hewson, Steele, et al., 2006; Wang et al., 2017). The literature mentions that the recycling of dissolved organic carbon and nutrients are the main ecosystem functions affected, directly impacting primary production and food webs (Bastida et al., 2016; Fontes et al., 2018; Hewson, Steele, et al., 2006; Tsementzi et al., 2016; Wang et al., 2017). However, bacterioplankton has shown a remarkable ability to respond to natural or anthropogenic environmental disturbances by restructuring community composition and community functioning (Allison & Martiny, 2008; Auladell et al., 2022; Baltar et al., 2015; Carlson & Hubert, 2019; Cram et al., 2015; Fontes et al., 2018; Lindh & Pinhassi, 2018; Lindh et al., 2016).

Crude oil exploration, extraction and transportation remain primary global economic activities (Carlson & Hubert, 2019; Lindh & Pinhassi, 2018; Morris et al., 2018). Therefore, the crude oil industry stands as a paramount source of pollution and disruption for marine ecosystems, given that most crude oil reservoirs are in marine environments (Bastida et al., 2016; Carlson & Hubert, 2019; Lindh & Pinhassi, 2018; Morris et al., 2018; Wang et al., 2017). The Gulf of Mexico (GoM), spanning approximately 1,600,000 km^2^, formed in the late Jurassic period, fostering crude oil seeps (Godoy et al., 2018; Peters et al., 2021). Reports from 2017 estimate that the GoM's crude oil production (Mexico + USA) ranges between 1.2 and 1.6 billion barrels yearly, primarily extracted in the Northern Gulf of Mexico (nGoM) (Peters et al., 2021). Hence, a constant need for environmental monitoring was established and after DeepWater Horizon (DWH) oil spill, the nGoM became a primordial study area to understand the environmental impact. As a result, the nGoM stands out as the most extensively studied oligotrophic ecosystem concerning the bacterial community's composition and its relationship with the environment (Rodríguez, Loza, et al., 2021).

A study conducted before the DWH oil spill in 2010 revealed an association between bacterioplankton composition in the nGoM and depth (King et al., 2013). In the surface waters at depths between 2 and 5 m, the bacterial community consisted of members from *Alpha‐proteobacteria*, *Bacteroidetes*, *Actinobacteria* and *Verrucomicrobia*. While, *Cyanobacteria*, *Alpha‐* and *Gamma‐proteobacteria* were the dominant classes at depths of ≤100 m. Moreover *Gamma‐* and *Delta‐proteobacteria* classes exhibited a positive correlation with depth (King et al., 2013). At depths >120, Archea had a substantial representation, where *Thaumarchaea* was the most abundant class (King et al., 2013). Observations between 2010 and 2016 revealed that the plume from the DWH crude oil spill led to an increase in the abundance of bacterial groups (e.g., *Marinobacter*, *Alcanivorax*, *Colwellia* and *Cycloclasticus*) with the capacity to degrade hydrocarbons (Kimes et al., 2014; Marietou et al., 2018). During this period, approximately 1600 genes related to hydrocarbon degradation were detected in the water column, where the gene expression was dominated by those related to monooxygenase enzyme production (Easson & Lopez, 2019; Kimes et al., 2014; Marietou et al., 2018; Mason et al., 2016). However, a recent study conducted in the epipelagic zone (0 to 200 m deep) mentioned that the bacterial dynamics and composition associated with the water column of nGoM are returning to the original conditions previous to the oil spill (Soto et al., 2014).

The Southern Gulf of Mexico (sGoM) relies heavily on oil‐related activities, such as extraction and transport, constituting one of the primary industries in the region (Cadena et al., 2019; Pardo & Gutiérrez, 2021). Unlike the nGoM, where numerous studies have focused on characterising microbial across various ecosystems and their potential responses to crude oil pollution, there has been limited attention dedicated to this aspect in the sGoM (Raggi et al., 2020; Rodríguez, Durán, et al., 2021). In a previous study conducted in the Perdido Fold Belt (nGoM) and Campeche Knolls (sGoM) areas, hydrocarbon degradation genes were detected in both regions. However, the microbial diversity and structure of bacterial communities were different (Raggi et al., 2020). Obtaining information on the structure and composition of microbial communities is essential for designing experiments that can predict the effects of oil concentration on biomass growth and the biodegradation of oil. This information is particularly relevant for developing effective bioremediation techniques (Denis et al., 2017).

In the Yucatan Shelf (YS) oceanic waters, microbial communities remain largely uncharacterized. Due to its proximity to oil and gas‐producing areas and its role as a transportation zone, the YS is susceptible to disturbances (Pardo & Gutiérrez, 2021; Peters et al., 2021). Therefore, the detection and monitoring of possible hydrocarbon‐degrading groups are essential for this region. As a result, our study aims to contribute to the characterisation of bacterial communities and the understanding of relationships between the native bacterial community and the physicochemical parameters in the sGoM water column. We particularly emphasise the presence of hydrocarbon‐degrading bacteria that may respond to oil contamination events in the YS area.

## EXPERIMENTAL PROCEDURES

### Study site and environmental variables

**FIGURE 1:**
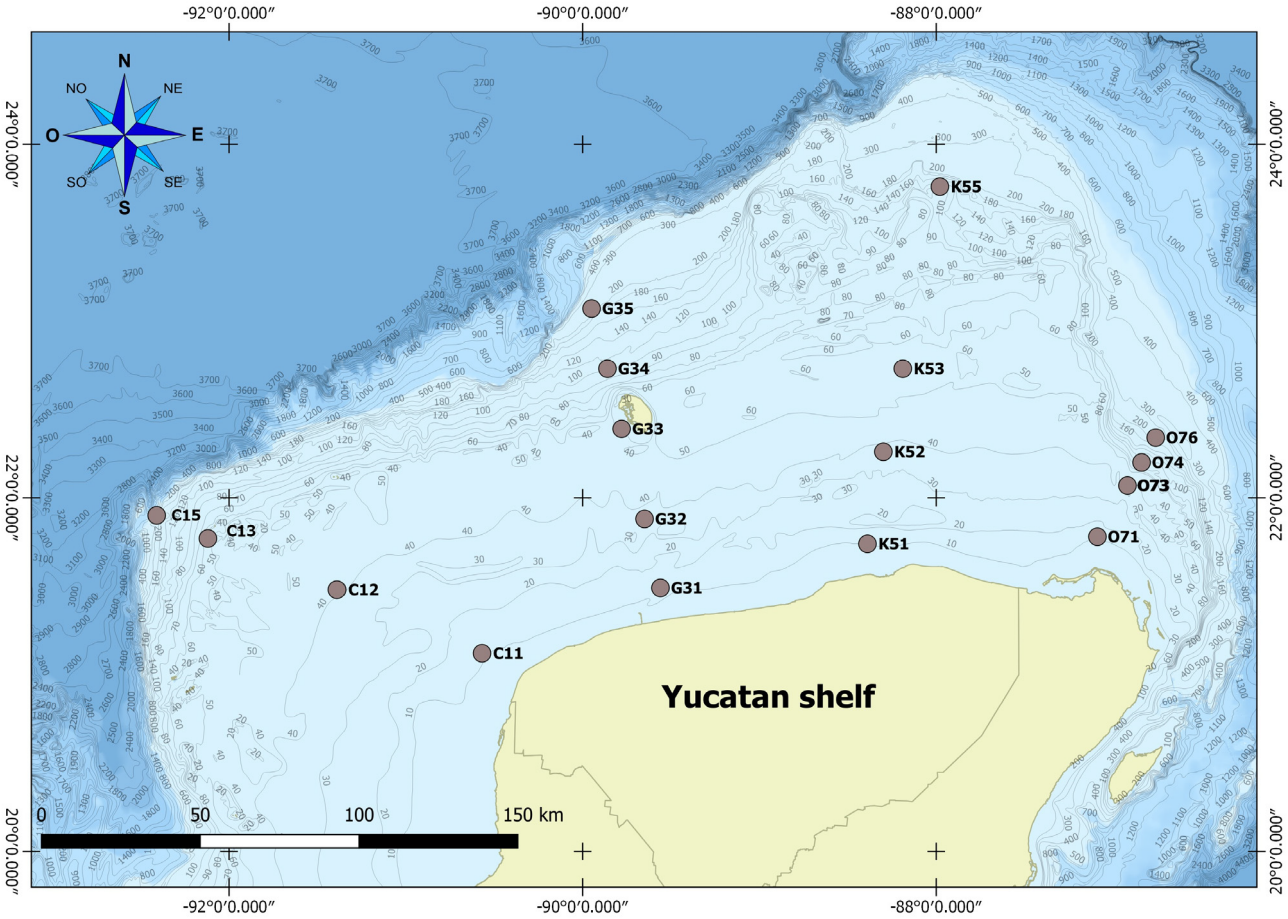
Study area and distribution of sampling sites.

Surface and bottom water samples were collected between August 23 and September 10, 2016, from four transects (C, G, K, and O), including four sites per transect in a depth gradient (Table 1) respect to sea flour, during the GOMEX V oceanographic campaign in the Yucatan shelf area in the sGoM (Figure 1). Surface water was collected at 5 m depth using a Van Dorn bottle (10 L) while bottom water was collected at ≈5 m before seabed using a rosette with 12 Niskin bottles attached. Salinity, temperature, chlorophyll‐a (*Chl‐a*.), dissolved oxygen (D.O.), and pH at each sampling point were measured employing a CTD seabird 9plus. Subsamples from the Van Dorn and Niskin bottles in sterile 1 L bottles for inorganic nutrients were stored at −20°C until analysis. Nitrate (NO^3^), orthophosphate (PO^4^), and silicic acid (SiO^4^) analyses were carried out according to Strickland and Parsons (1972) method. Lectures were performed using an Agilent (Cary 60 UV‐VIS) spectrophotometer. The detection limits for each inorganic nutrient were as follows: 0.03 μmol L^−1^ for nitrates, 0.01 μmol L^−1^ for nitrites, 0.02 μmol L^−1^ for phosphates, and 0.05 μmol L^−1^ for silicates.

**TABLE 1:** Geographic location data for the stations and transects.

Station	Latitude	Longitude	Max depth (m)	Distance stations (nm)	Transect length (nm)	Dist. transects (nm)
C11	21.12	−90.57	16	‐	111.21	C‐G 172
C12	21.48	−91.39	37	50.32		
C13	21.77	−92.12	49	30.74		
C15	21.90	−92.41	150	30.15		
G31	21.49	−89.56	10	‐	75.51	
G32	21.88	−89.65	27	24		
G33	22.39	−89.78	44	31.21		G‐K 139
G34	22.73	−89.86	85	20.3		
G35	23.07	−89.95	143	‐		
K51	21.74	−88.39	16	31.42	121.52	
K52	22.26	−88.30	32	27.8		
K53	22.73	−88.19	45	62.3		
K55	23.76	−87.98	103	‐		
O71	21.78	−87.09	17	19.57	37.91	K‐O 125
O73	22.07	−86.92	24	9.04		
O74	22.20	−86.84	65	9.3		
O76	22.34	−86.76	179	‐		

*Note*: Both latitude and longitude are given in minutes and seconds. Depth is given in meters (m), and distances are provided in nautical miles (nm). The distance between transects (Dist. Transects) was measured from the stations that were furthest apart. The distance between stations was measured within each transect.

### Quantification of hydrocarbons in the water column (surface and bottom water)

Four litres of seawater from the Van Dor bottles (surface) and rosette (bottom) at each station were kept in 4 L amber bottles and refrigerated (4°C) until hydrocarbon analysis. A liquid–liquid extraction (1:1) of hydrocarbons was carried out directly in the bottles. Each bottle was added 50 mL of dichloromethane and stirred for 10 min three times. After the last agitation, it was allowed to stand until the formation of the two phases. Then, the dichloromethane phase (aqueous lower) was retrieved using a volumetric pipette. The retrieved dichloromethane extract was deposited in a 125 mL amber bottle and maintained at −4°C until analysis. The dichloromethane extracts were concentrated and reduced to a volume of 10 mL with the help of a rotary evaporator. Nitrogen gas was used to continue reducing the extracts to a volume of 0.5 mL.

Hydrocarbon concentration in the water column was measured as the total petroleum hydrocarbon (TPH), determined as the sum of aliphatic compounds, aromatic compounds and unresolved complex mixture (UCM) concentrations. Aliphatic compounds and UCM were analysed using a Gas Chromatograph Agilent 7683B auto‐injector (Agilent Technologies, USA) equipped with a flame ionisation detector (GC‐FID) and a J &W Scientific DB‐5 capillary column (30 m × 0.32 mm × 0.25 μm). FID conditions for the ignition were an H_2_ flow of 30 mL min^−1^ and an airflow of 300 mL min^−1^. Helium at 25 mL min^−1^ was used as the makeup flow. Samples (1 μL) were injected in splitless mode at 290°C, using helium as the carrier gas (flow rate of 1.5 mL min^−1^). Compounds were analysed with the following program: start temperature 50°C for 1 min, heating to 300°C for 5 min. Aromatic compounds were analysed on a Perkin Elmer Clarus 500 gas chromatograph coupled to an ion trap mass spectrometer (GC‐MS). An HP‐5MS fused‐silica column (30 m × 0.25 mm × 0.25 μm) was used. The mass spectrometer detector operated in the selective ion monitoring (SIM) mode for quantification. The chromatographic conditions were as follows: carrier gas, helium (1.1 mL min^−1^); injection mode, splitless; injector and detector temperatures, 270 and 200°C, respectively. For the analysis of specific polycyclic aromatic hydrocarbons (PAHs), the following program was used: 70°C for 3.5 min; ramp at 10°C min^−1^ to 300°C; and hold for 5 min. Hydrocarbon concentrations were determined according to standards, including the 40147‐U Supelco C8‐C40 alkanes calibration standard (Sigma‐Aldrich, Bornem, Belgium) and CUS‐19547 aromatic standards (Ultra Scientific, North Kingstown, RI, USA).

### Estimation of culturable hydrocarbonoclastic bacteria from water column

To estimate the culturable hydrocarbonoclastic bacteria (CHB) per sample site, water samples (150 mL in sterile bottles) from the surface and bottom water were refrigerated at 4°C until analysis. The determination was performed using 10 μL sterile tubes, each containing 5 mL of Bushnell‐Hass (B‐H) medium (Difco, Livonia, Michigan, USA) conditioned with 2% NaCl, pH 7. Test tubes were inoculated with 10 mL of seawater from the campaign, light crude oil (0.178 mg L^−1^) as the sole carbon source, and resazurin as an indicator of bacterial growth (García et al., 2018; Lizárraga et al., 1991). The most probable number (MPN) method reported by Rice and Hemmingsen (1997) and Lizárraga et al. (1991) was used for the estimations.

### Water column DNA extractions and 16S rRNA sequencing

Eight litres of seawater were collected in Nalgene containers from each station and depth and filtered with a peristaltic pump through a 0.22 μm Sterivex filtration unit (Millipore Corp., Bedford, MA, USA). After filtering the seawater, the excess water was removed from the filtration units using a syringe (60 mL). Each Sterivex unit was plugged, labelled, wrapped in parafilm, and preserved in liquid nitrogen until arrival at the laboratory. Once in the laboratory, the Sterivex units were maintained at −80°C until analysis. DNA extraction was performed following the instructions of the PowerWater Sterivex DNA Isolation Kit (Qiagen, Germantown, MD). The quality of DNA extraction was assessed by electrophoresis (1% agarose gel). The amplification of the hypervariable V3 and V4 region of 16S rRNA was conducted using the primers (S‐D‐Bact‐0341‐b‐S‐17, 5′‐CCTACGGGNGGCWGCAG‐3′ and S‐D‐Bact‐0785‐a‐A‐21, 5′‐GACTACHVGGGTATCTAATCC‐3′) and the conditions suggested by Klindworth et al. (2013). Amplicons were purified with AMPure XP beads (Beckman Coulter Genomics, Brea, CA) and indexed using the Nextera XT kit (Illumina, San Diego, CA, USA), according to the library preparation protocol recommended by the manufacturer. Indexed PCR products were purified and quantified with a Qubit® 3.0 Fluorometer using the Qubit dsDNA HS Assay Kit (Life Technologies, Carlsbad, CA, USA). Amplicon size was verified by capillary electrophoresis at QIAxcel Advanced (QIAGEN, Valencia, CA, USA). Individual amplicons were diluted in 10 mM Tris (pH 8.5) and pulled at equimolar concentrations (4 nM). Sequencing was carried out in CINVESTAV‐Mérida using an Illumina‐MiSeq platform (Illumina, San Diego, CA, USA), with the MiSeq reagent kit V3 (2 × 300), following the manufacturer's recommendations.

### Quantification of the *alkB* gene

The quantification of the *alkB* gene, related to first‐step hydroxylases involved in the metabolism of alkanes, was performed on a Rotor‐Gene Q System (Qiagen, Hilden, Germany) with the primers and the protocol previously reported by Uribe et al., (2019), with coefficient of determination (*r* ^2^) of 0.98 and PCR amplification efficiency of 98.5%.

### Data, bioinformatic and statistical analyses

For the bacterial community analysis, the demultiplexed paired‐end reads (2 × 300) in the fastq format were processed with the QIIME2 (2019.1) pipeline (Caporaso et al., 2010). The error correction and denoising to resolve the amplicon sequence variants (ASVs) were performed with the DADA2 plugin (Callahan et al., 2016, 2017). The length of sequences was 250 bp after trimming. Chimeras were removed with the ‘consensus’ method. The representative ASVs were taxonomically assigned with the V‐SEARCH consensus taxonomy classifier plugin (Rognes et al., 2016) using the SILVA database (v.128) as a reference. A phylogenetic tree was built with the reference ASVs with the FastTree algorithm (Price et al., 2010). This tree was utilised for calculating the dissimilarity matrix, and among the resulting dissimilarity matrices created. To analyse the samples, normalisation was performed with phyloseq_transform_css function, then transformed to percentages to obtain relative abundances. The abundance table was exported to the R environment, and the statistical analysis and visualisation were performed with the phyloseq (McMurdie & Phyloseq, 2013), vegan 40 and ggplot2 (Oksanen, 2015)  libraries. Pairwise dissimilarities were calculated using UniFrac (Unweighted) metrics (Lozupone et al., 2011; Wilkinson, 2011). Each resulting dissimilarity matrix was used to visualise differences in the samples through a dendrogram (Podani & Schmera, 2006). The metadata matrix was constructed with the physicochemical parameters measured during the GOMEX V oceanographic campaign of each variable by station and depth; each parameter was used as an independent variable. The variation in bacterioplankton diversity and structure was assessed using the PERMANOVA adonis function of the vegan package (Oksanen et al., 2008;  Wilkinson, 2011). Alpha diversity was estimated with the vegan library (R environment). Through a SIMPER test (similarity percent analysis) the ASVs that contribute to the formation of clusters or groups were detected. To observe if any of the variables contributed to the formation of these groups, one‐way ANOVA was performed. Finally, a Venn diagram was made among the ASVs obtained from the SIMPER analysis corresponding to the four selected transects.

## RESULTS

### Physicochemical determinations for the water column of Yucatan shelf area (sGoM)

The physicochemical parameters measured in this study are showed in Table 2. The minimum value (stations from surface water G35, K51, K53, O73 and O74) for *Chl‐a* was 0.2 mg m^3^, however, 50% of the measures of this parameter were above of 0.50 mg m^3^, and the maximum value (stations from bottom water G33, K53, O74 and O71) registered was 2 mg m^3^ (for full measures, see Table S1).

**TABLE 2:** Physicochemical variables from the surface (5 m depth) and bottom water samples (from 10 to 176 m depth), including standard deviation (SD).

Variable	Mean (SD)	Mode	Median	Minimum range	Maximum range
Chlorophyl a (mg m^3^)	0.60 (±0.50)	0.3	0.50	0.20	2
Dissolved oxygen (mg L^−1^)	4.24 (±0.83)	4.03	4.33	2.06	5.93
Salinity (g Kg^−1^)	36.49 (±0.06)	36.52	36.49	36.41	36.78
pH	8.5 (±0.9)	9.09	9.01	6.80	9.14
Temperature (°C)	25.71 (±4.31)	N/D	26.02	16.65	30.68
NO^3^ (μmol L^−1^)	2.84 (±4.11)	N/D	0.80	0.23	13.90
PO^4^ (μmol L^−1^)	0.11 (±0.15)	N/D	0.07	0.03	0.73
SiO^3^ (μmol L^−1^)	2.57 (±0.11)	1.87	2.42	1.04	6.47

Abbreviations: N/D, no detected; NO^3^, nitrates; PO^4^, orthophosphates; SiO^3^, silicates.

The bottom water samples from the deepest stations (80 to 180 m) presented the lowest concentrations (2.06 to 3.3 mg L^−1^, stations C15, G35, K55 and O76) of D.O. The frequently occurring D.O. concentration in the water column was 4.03 mg L^−1^, the maximum D.O. level observed was 4.85 3 mg L^−1^ at station K51 (surface water). The average salinity was 36.49 (±0.06) Kg^−1^. The temperature range was from 16.65 (C15 bottom water) to 30.68°C (G35 surface water). As for pH, the minimum value was 6.80 (O76 bottom water), and the maximum was 9.14 (C12 bottom water), with a modal value of 9.09 and 9.01 for the median (Table 2 and for full measures Table S2).

Regarding nutrients (for full measures, see Table S2), nitrates (NO^3^) showed a wide variation ranging from 0.23 (K53 surface water) to 13.90 (G35 bottom water) μmol L^−1^, with a central value of 0.803 μmol L^−1^. Silicates (SiO^3^) displayed a broad range between the minimum value observed in the surface water of the station O74 (1.04 μmol L^−1^) and the maximum value (6.47 μmol L^−1^) in station G35 bottom water. In contrast, the orthophosphates (PO^4^) concentrations range was low (from 0.03‐C15 surface water to 0.73‐O76 bottom water μmol L^−1^), with a median value of 0.070 μmol L^−1^ (Table 2).

### Quantification of hydrocarbons, CHB and *alkB* gene for water column of Yucatan shelf area (sGoM)

TPH in the surface water (Table 3) exhibited a range from 0 to 0.72 μg L^−1^, with stations G31 and O71 displaying the highest values. Comparable values (ranging from 0.12 to 0.86 μg L^−1^) were observed in the bottom water (Table 3). At both depths, the minimum count of colony‐forming units (CFUs) was 40 CFU mL^−1^, while the maximum was 24,000 CFU mL^−1^ (Table 3). The surface water displayed elevated values (ranging from 68,226.52 to 126,189,398.35 copies/mL) of *alkB* gene copies per mL (Table 2). Although the linear regression analysis (Table S2) demonstrated no statistical association between TPHs and CFUs or the *alkB* gene at any depth, stations with high CFU values exhibited the greatest number of *alkB* gene copies (Table 3).

**TABLE 3:** Values of hydrocarbons (TPH's), culturable hydrocarbonoclastic bacteria (CHB), and *alkB* gene quantifications in Yucatan Shelf's water column.

Station	Depth (m)	TPHs (μg L^−1^)	CHB (CFU mL^−1^)	*alkB* gene (copies mL^−1^)
C11	5	0.28	40	76,820.97 (±585.86)
C11	16	0.68	110	10,947.78 (±2434.30)
C12	5	0.09	2400	41,579.37 (±8573.89)
C12	37	0.12	1500	74,266.44 (±16,462.90)
C13	5	0.08	430	53,723.92 (±1796.18)
C13	49	0.15	100	34,690.03 (±10,289.1)
C15	5	0.00	2400	83,005.78 (±2815.28)
C15	150	0.48	110	57,159.43 (±6558.0)
G31	5	0.72	2550	174,510.05 (±18,056.40)
G31	10	0.06	210	84,702.54 (±127.71)
G32	5	0.54	2200	170,455.61 (±28,238.10)
G32	27	0.78	460	ND
G33	5	0.35	150	430,467.52 (±86,993.28)
G33	44	0.57	100	28,024.58 (±1447.29)
G34	5	0.06	150	70,560.75 (±2666.08)
G34	85	0.86	110	ND
G35	5	0.00	30	68,266.52 (±401.78)
G35	143	0.06	1200	32,244.95 (±3040.04)
K51	5	0.13	2400	82,974.42 (±10,868.14)
K51	16	0.06	2400	42,661.60 (±14,443.65)
K52	5	0.10	2550	189,610.80 (±77,684.08)
K52	32	0.14	40	653.17 (±4958.75)
K53	5	0.16	1100	21,079.43 (±5388.66)
K53	45	0.22	2400	39,809.72 (±2107.26)
K55	5	0.31	4600	257,529.84 (±3397.33)
K55	103	0.25	4300	848,162.75 (±2050.06)
O71	5	0.53	4200	3,277,678.87 (±245,622)
O71	17	0.40	2400	651,682.19 (±92,825.60)
O73	5	0.30	4250	903,168.54 (±78,200.20)
O73	24	0.74	1500	9016.46 (±9083.7)
O74	5	0.54	750	95,556.21 (±1908.80)
O74	65	0.62	110	ND
O76	5	0.71	24,000	126,189,389.35 (±1,402,923.08)
O76	179	0.66	100	ND

*Note*: Standard deviation (SD) for *alkB* gene is provided in parentheses.

### Bacterioplankton community structure and composition from water column of Yucatan shelf area (sGoM)

**FIGURE 2:**
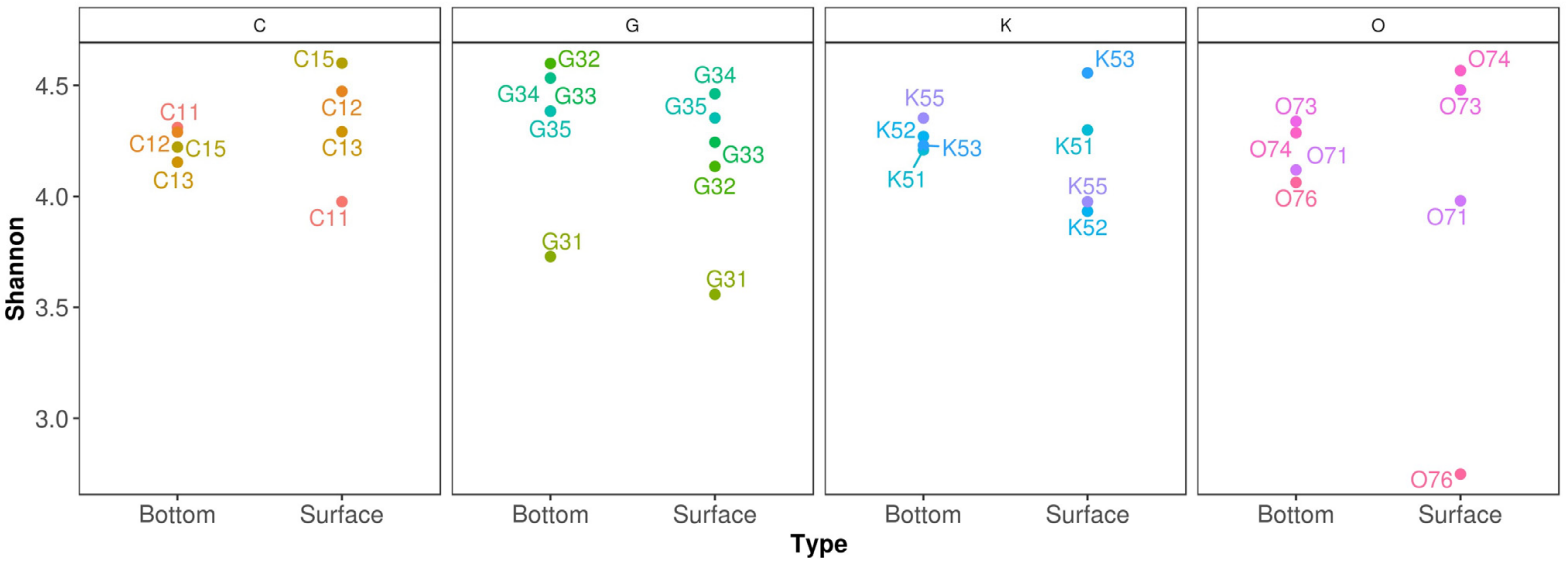
Shannon diversity of the sampled stations from the surface and bottom water. The colours in the figure are used to distinguish the values obtained in the different stations sampled in each transect (C, G, K, O).

Figure 2 displays Shannon's diversity for each transect and water layer. Diversity values generally ranged between 3.5 and 5.52 in both water layers, with a remarkably low value in site O76 from surface water (2.63). The highest values occurred in surface water sites C15, K53 and O74, G32 and G34 of bottom water (Figure 2). However, there was no statistical association (PERMANOVA: *R* ^2^ = 0.03, *p* = 0.37) between diversity and depth.

**FIGURE 3:**
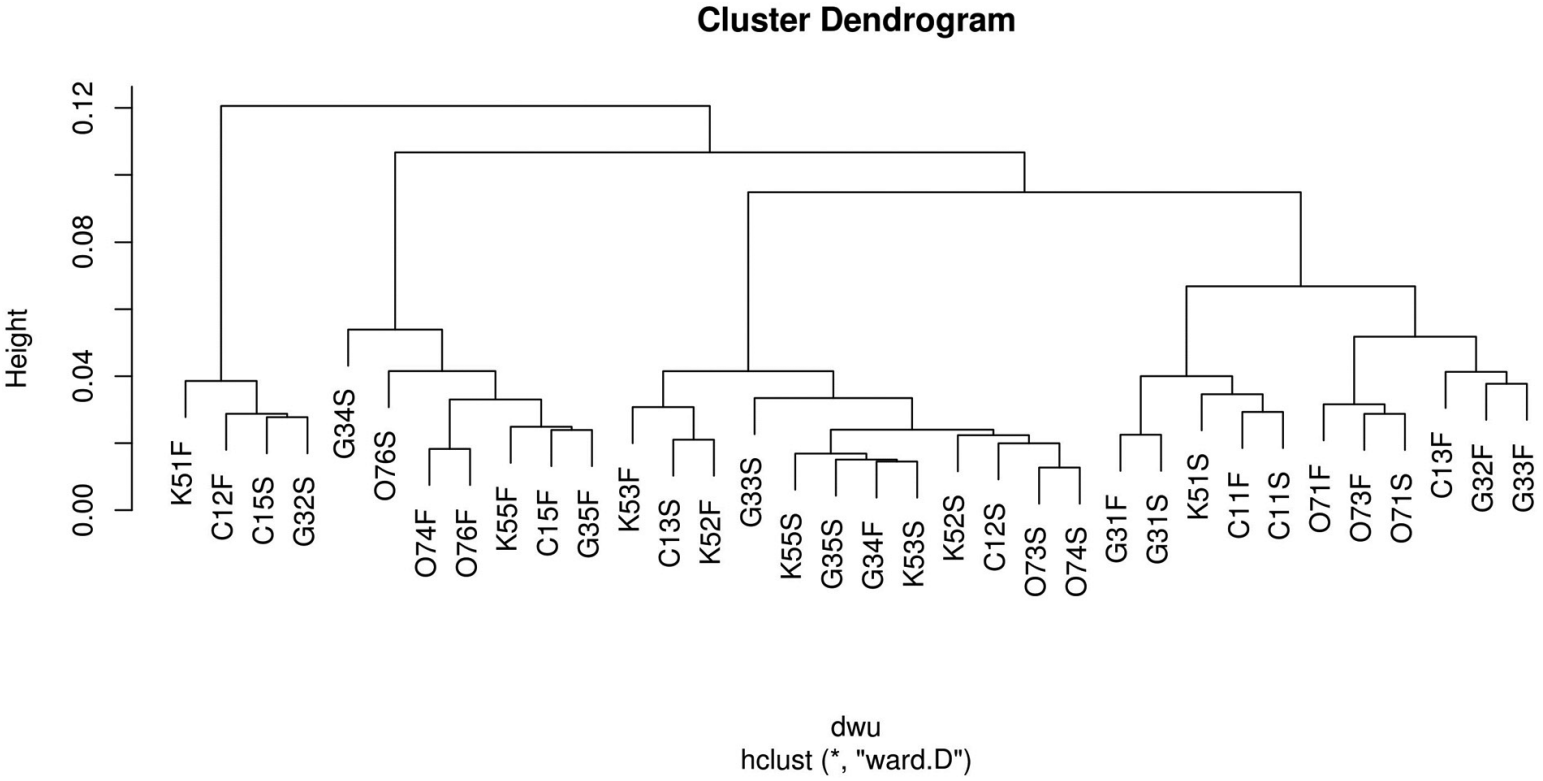
Dendrogram (distance: Unweighted Unifrac) of the clusters obtained from the bacterial community similarities in surface and bottom water samples. The cut‐off line is set at a distance of 0.08.

The stations were grouped into four clusters (cut off‐line at 0.08; Figure 3) based on the unweighted Unifrac distance. Within all clusters (DPCoA analysis is shown in Figure S1), samples from both the surface and bottom water were present. PERMANOVA analysis indicated that the primary variable explaining the community structure changes is the depth gradient of the ocean shelf (*R* ^2^ = 0.63, *p* = 0.011). Other influencing factors include dissolved oxygen (D.O., *R* ^2^ = 0.056, *p* = 0.016), *Chl‐a* values (*R* ^2^ = 0.046, *p* = 0.045), and the station locations (*R* ^2^ = 0.56, *p* = 0.002) which contribute to variations in the bacterioplankton structure and composition (Figure 3).

**FIGURE 4:**
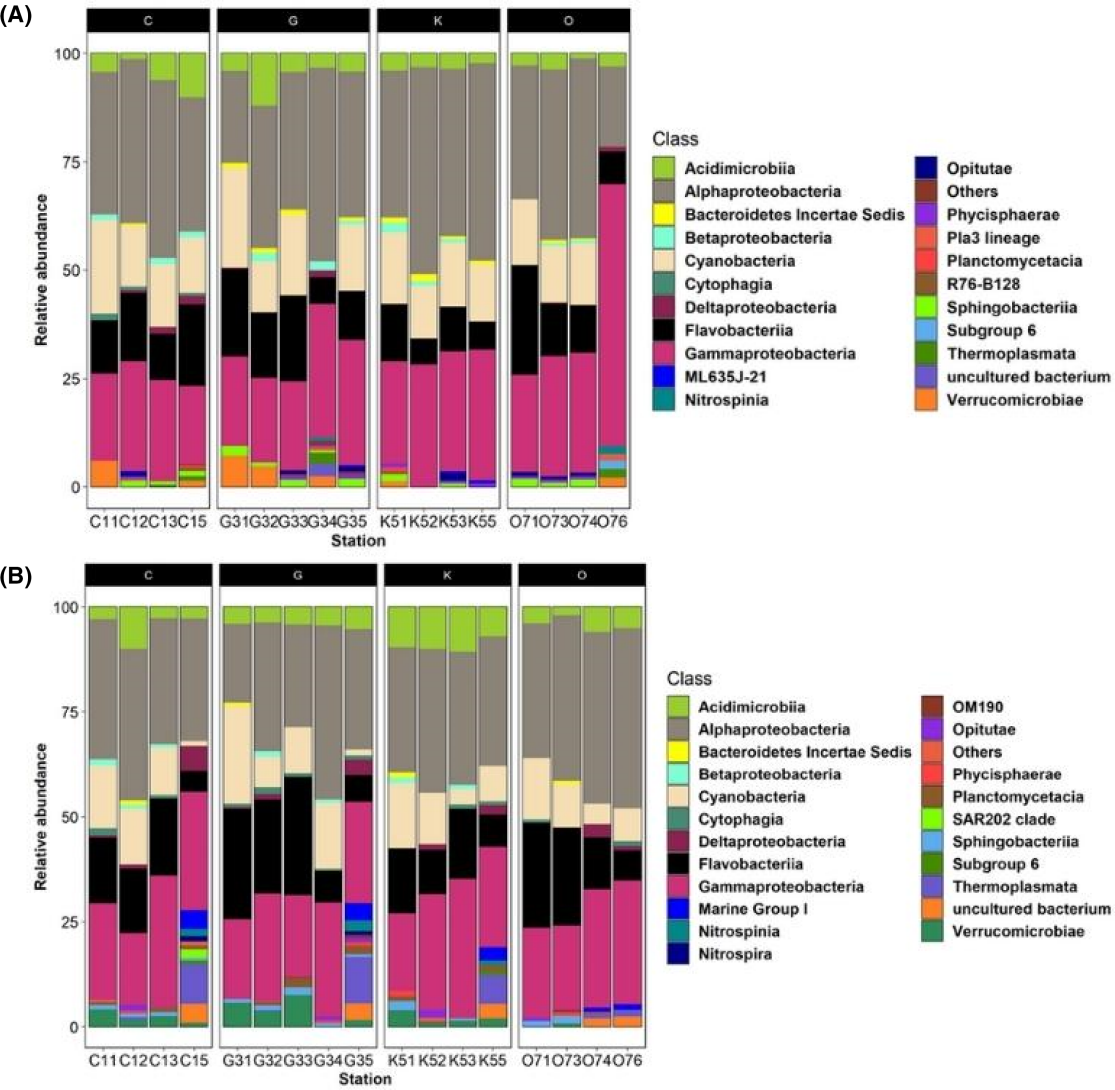
Relative abundances graphics of the bacterial community from the water column of the sGoM at the class level. Where subsection (A) corresponds to surface water (5 m depth) and subsection (B) to bottom water (water samples were taken 5 m before the maximum depth of each station).

The composition of the dominant ASVs was similar across all sampled stations and depths (surface and bottom water). The dominant classes were *Alpha*‐ and *Gamma‐proteobacteria* (Figure 4A,B). The relative abundances for *Alpha‐proteobacteria* ranged between 25% (Station G31) and 45% (Station K52) in surface water (Figure 4A), while in bottom water (Figure 4B), the highest abundance for this group was 48% (Station O76). In contrast, the *Gamma‐proteobacteria* class in surface water (Figure 4A) exhibited values between 20% (Stations C11 and C15) and 35% (Stations C12, G34, G35, K52, K55, O73 and O74) in most stations, except for station O76, where it accounted for 60% of the relative abundance. However, in the bottom water (Figure 4B), the abundance of *Gamma‐proteobacteria* ranged from 18% (Stations C12, G31 and K51) to 33% (Stations C13 and K53).

*Cyanobacteria*, *Flavobacteriia*, *Acidimicrobiia*, *Sphingobacteria* and *Verrucomicrobiae*, constituted the most abundant groups (Figure 4A,B). The classes *Flavobacteriia* (15% to 25%), *Acidimicrobiia* (5% to 22%), and *Sphingobacteriia* (5% to 8%) showed very similar relative abundances between surface water and bottom water (Figure 4A,B). In contrast, the relative abundances of *Verrucomicrobiae* were higher in the bottom water (5% to 12%).

**FIGURE 5:**
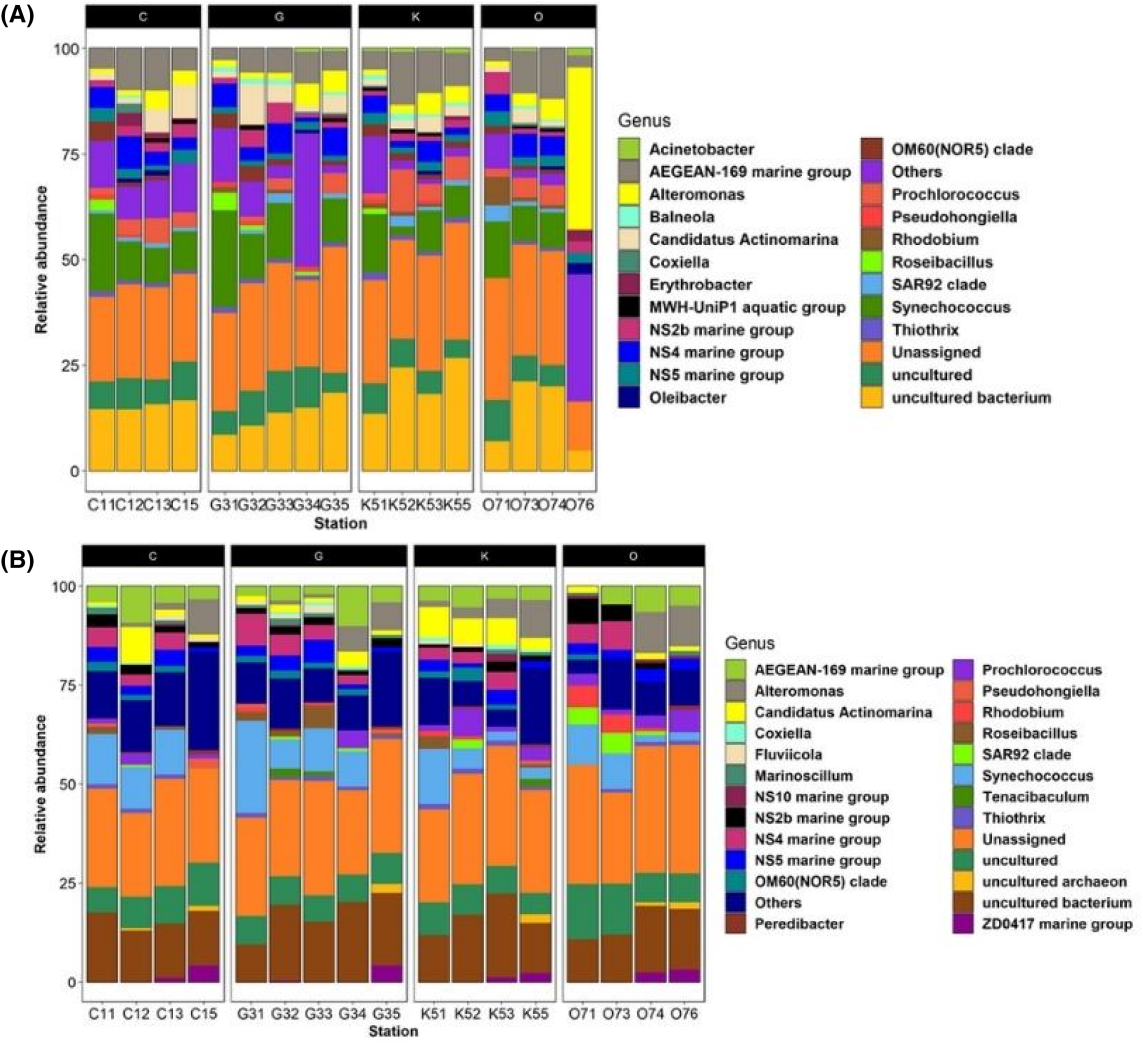
Relative abundances graphics of the bacterial community from the water column of the sGoM at the genus level. Where subsection (A) corresponds to surface water (5 m depth) and subsection (B) to bottom water (water samples were taken 5 m before the maximum depth of each station).

In all sampled stations, at both depths (surface and bottom), the bacterioplankton composition at the genus level (Figure 5A,B) shows that more than 63% of the relative abundances belong to the groups of ASVs classified as unassigned, unclassified bacteria, and genera with relative abundances ≤5%. Genera with relative abundances ≤5% were categorised as ‘others’ in Figure 5A,B. Despite, *Alteromonas*, *Synechococcus*, *Phrochlorococcus*, *Rhodobium*, *Thiothrix*, *Pseudohongiela*, *Coxiella*, NS2b marine group, NS4 marine group, NS5 marine group, clade OM60(NOR5), clade SAR92 and *Candidatus Actinomarina* were present in both depths, Only *Alteromonas*, *Synechococcus*, *Candidatus* Actinomarina, and the NS5 marine group, were present in all stations (Figure 5A,B).

*Alteromonas*' relative abundances ranged from around 8% to 15% of the composition in the surface water samples, except for station O76, where it reached 60% (Figure 5A). In the bottom water (Figure 5B), the relative abundance values ranged between 7% and 17%. However, at stations C11 (16 m depth), G31 (10 m depth), O71 (17 m depth) and O73 (24 m depth), the relative abundances of this group were less than 5% (Figure 5B).

The genera uniquely found in surface water (Figure 5A) were *Acinetobacter* with an abundance of 5%, *Balneola* (5% to 8%), *Erythrobacter* (5% to 10%), MWH‐UniP1 aquatic group (5% to 7%), *Oleibacter* (5% to 10%) and *Roseibacillus* (5% to 13%). In the case of bottom water, the exclusive genera (Figure 7) were *Fluviicola* (5% to 7%), *Marinoscillum* (7% to 12%), *Peredibacter* (5% to 14%), *Tenacibaculum* (5% to 8%), and ZD0417 marine group (5% to 12%).

### Similarity percentage analysis (SIMPER) and Venn diagram of the bacterioplankton community of the Yucatan shelf area (sGoM)

**FIGURE 6:**
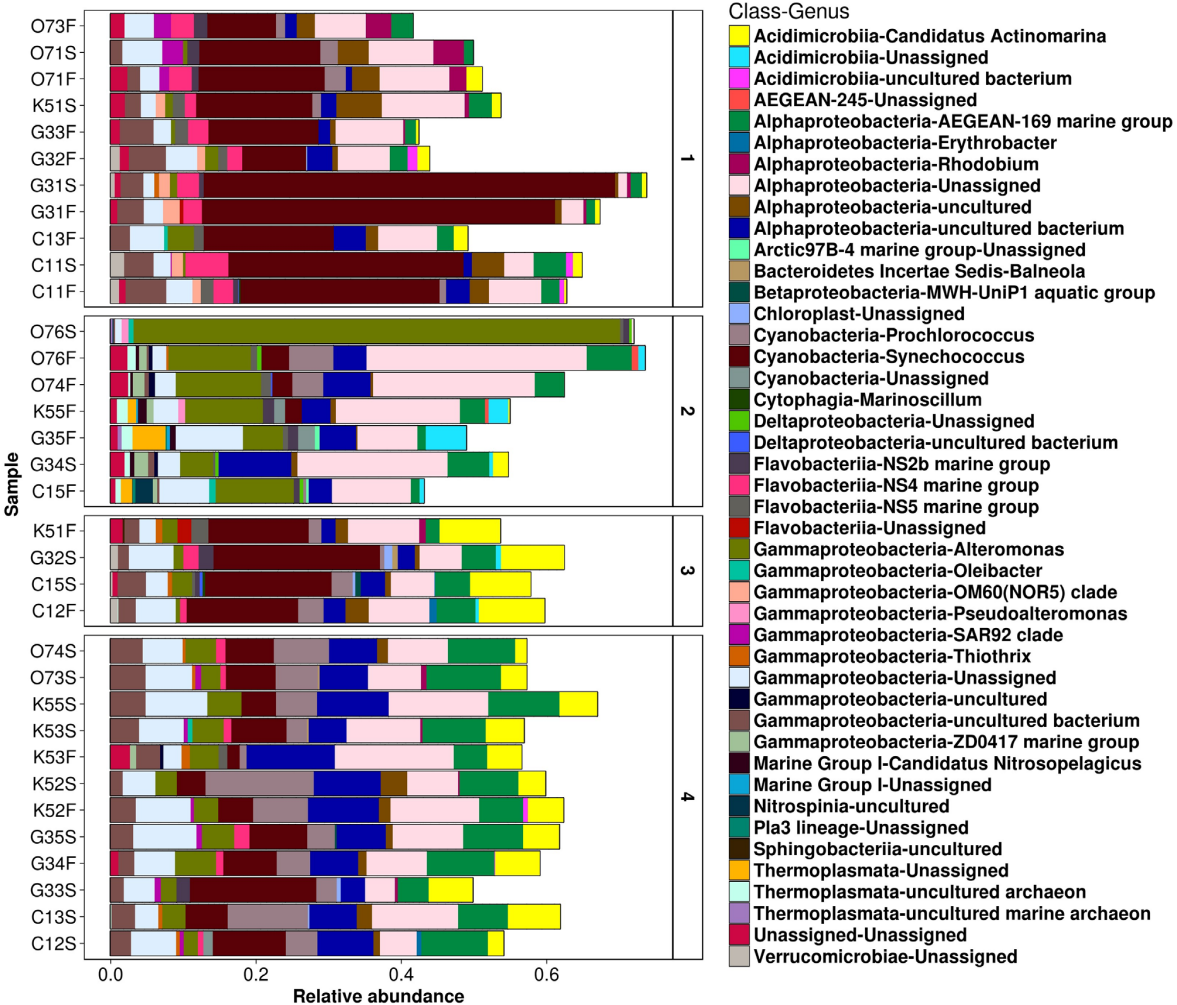
Results of the SIMPER analysis. The graphic displays the similarity percentages of the bacterioplankton community composition for each cluster observed in the dendrogram. The cluster number is indicated to the right of the graphs using Arabic numerals. The *x*‐axis shows the scale of relative abundances, ranging from zero to one (where 1 = 100%).

The results of the SIMPER analysis conducted on the four clusters obtained from the dendrogram (Figure 3) are presented in Figure 6. This analysis allows identifying the characteristic composition of each group (Figure 6). For instance, the first cluster is distinguished by displaying the highest relative abundances of the genera *Synechococcus* (*Cyanobacteria*), marine group NS4 (*Flavobacteriia*), Clade OM60 (*Gamma‐proteobacteria*), and an unassigned genus belonging to *Verrucomicrobiiae*. Another characteristic of this cluster is the lowest abundances of two unassigned genera from the *Alpha‐proteobacteria* class, *Candidatus Actinomarina* (*Acidimicrobiia*), and the marine group AGEAN‐169 (*Alpha‐proteobacteria*). Among the samples of this cluster, three were surface stations (5 m depth) and five were bottom water, with the following depths 10, 16, 17, 24 and 44 m. the minimum diversity of this group was 3.66, and the maximum of 4.26 H′ (Figure 2).

The one‐way ANOVA analysis indicated a significant relationship between *Chl‐a* concentrations (*p* = 0.022—summary Table S3) and the grouping of samples based on the composition determined by the SIMPER analysis. Likewise, the multiple comparisons among the groups showed that cluster two differs (*p*_adj_ of 0.05—Table S4) from the rest of the groups (Figure 6). Cluster two was characterised by the lowest abundances of the *Cyanobacteria* members and by the presence of several unassigned genera belonging to the classes *Alpha*‐, *Gamma*‐, *Delta‐proteobacteria*, AGEAN‐245, the *lineage Pla3*, *Acidimicrobiia*, *Sphingobacteriia* and *Nitrospina*. In addition, the presence of *Oleibacter*, AGEAN‐169, and the highest relative abundances of *Alteromonas* (*Gamma‐proteobacteria*), the marine group ZD0417 (*Gamma‐proteobacteria*), as well as three unassigned genera of Archaea of the class *Thermoplasmata*. Most of the samples in group two were from bottom water, with the highest sampling depth found on the ocean shelf (between 65 and 179 m). Nonetheless, two samples (O76 and G34) were from surface water (Figure 6).

The characteristic community composition of clusters three (stations C12 and K51 from the bottom C15 and G35 from surface water station) and four (12 samples grouped, 10 from surface water and two from bottom water) were similar to that of cluster one (Figure 6). However, cluster three performed the highest abundances of *Candidatus Actinomarina*, while in cluster four, the highest abundances were for genera *Prochlorococcus* and AGEAN‐169. The main difference between the composition of clusters three and four with cluster one was the presence of the taxa *Thiothrix* (Figure 6).

**FIGURE 7:**
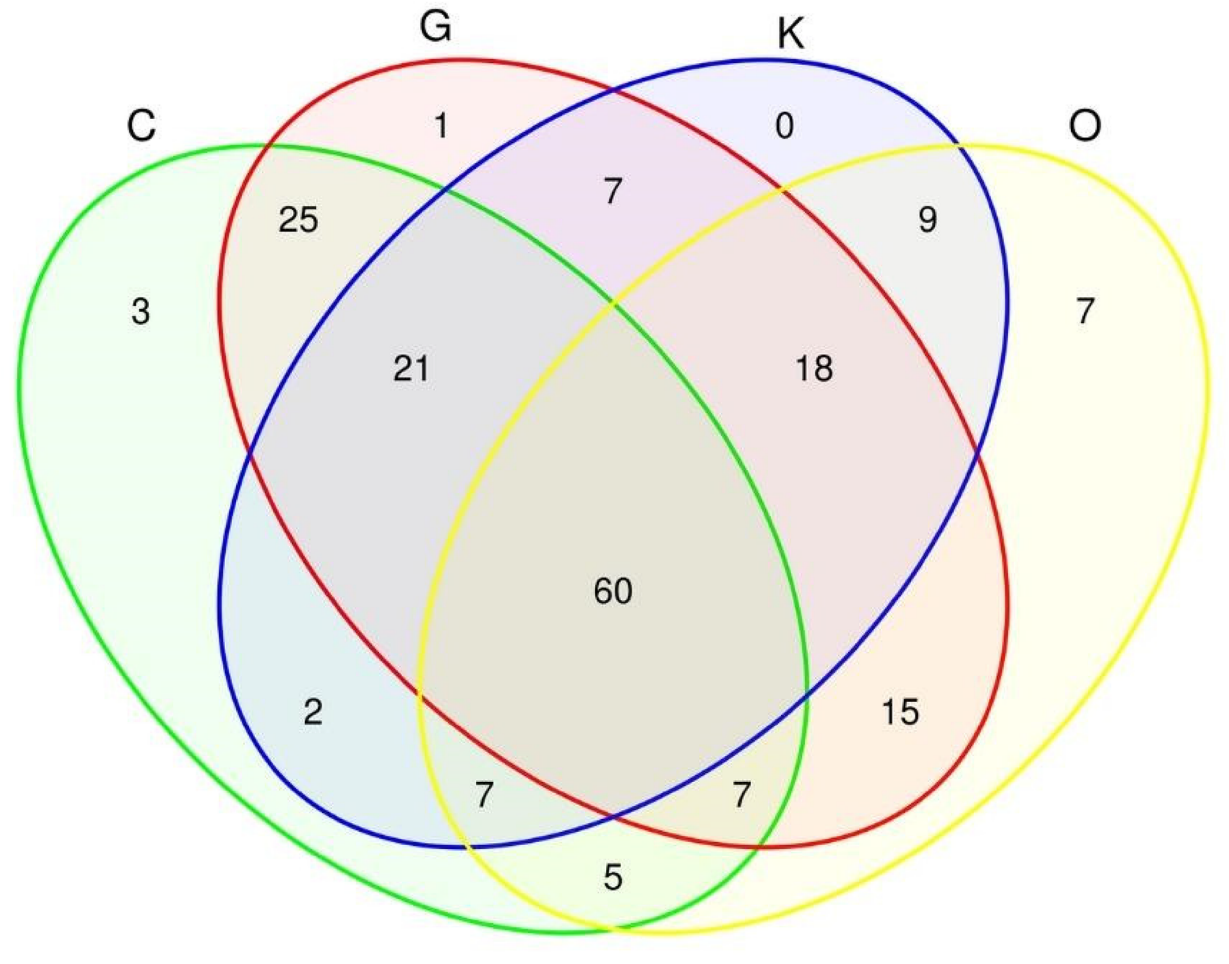
Venn diagram of the transects sampled in the sGoM, based on SIMPER analysis. Each transect is represented by a different colour: Transect C is green, G is red, K is blue and O is yellow. Arabic numbers within the diagram represent the number of taxa. Each intersection (overlap) of the colours indicates the number of shared taxa between the transects.

Despite the distance (Table 1) among the transects C, G, K and O (Figure 1), the bacterial community associated with the water column of the YS area is similar, as evidenced by the sharing of up to 60 taxa (Table S5), as shown in the Venn diagram (Figure 7). However, the largest overlaps are observed among transects C, G (25 taxa—Table S5), and K (21 taxa—Table S5), whereas transect O registered the most significant number of unshared taxa.

## DISCUSSION

### Bacterioplankton community composition, structure and environmental parameters

During the oceanographic campaign GOMEX V, the classes *Alpha*‐, *Gamma‐proteobacteria*, *Cyanobacteria, Flavobacteriia*, *Acidimicrobiia*, *Sphingobacteria* and *Verrucomicrobiae* (Figure 4A,B) were the representative groups in the water column of the sGoM. These taxa, except for *Flavobacteriia*, have been previously reported in the water column of non‐tropical marine environments (Auladell et al., 2022; Bunse & Pinhassi, 2017). As observed in previous studies of microbial ecology in marine ecosystems, the dominant bacterial groups in the sGoM were *Alpha*‐ and *Gamma‐proteobacteria* (Auladell et al., 2022; Bunse & Pinhassi, 2017). For *Flavobacteriia*, previous reports suggested that this group could be typical in tropical coastal areas (Enriquez et al., 2010; Kumagai et al., 2018; Mann et al., 2013; Pollet et al., 2018). Furthermore, the growth of the members of this group is benefited by the presence of high levels of irradiance due to the presence of the ‘PR’ gene, which serves as a sun shield (i.e., a protector against irradiance) and enables the conversion of sunlight into ATP (Mann et al., 2013). Previous solar irradiance conditions reported for the Yucatan Shelf area (Seo et al., 2017) lead us to conclude that this parameter promotes the growth of *Flavobacteriia* members in the water column of this region.

At the genus level, the presence of *Alteromonas, Synechococcus*, *Candidatus Actinomarina*, and NS5 marine group in all stations (Figure 5A,B) together with the hydrocarbon quantification (Table 3) suggest that the water column of the YS does not show signs of oil pollution. Previous studies have reported that *Candidatus Actinomarina*, NS5 marine group and *Synechococcus* are representative genera of coastal areas with low pollution concentrations (Caruso, 2020; Zhang et al., 2021).

In the case of the bacterioplankton of the Yucatan Shelf area (sGoM), PERMANOVA analysis indicates that depth (*R* ^2^ = 0.63, *p* = 0.011), dissolved oxygen (D.O.—*R* ^2^ = 0.056, *p* = 0.016) concentration, and *Chl‐a* (*R* ^2^ = 0.046, *p* = 0.0E45) values (Table S1) are the primary factors associated with changes in the structure and composition of the bacterial community in this region. These results support the notion that changes in bacterial communities in marine environments are associated with alterations and variability in physicochemical parameters such as temperature, salinity, pH, and nutrient concentrations, along with factors like season, light availability and water depth (Parada & Fuhrman, 2017).

Cluster two displayed significant differences from the other clusters. According to the SIMPER analysis (Figure 6), some of the characteristic taxa of cluster two included ASVs from the classes Marine Group I (Archaea), *Thermoplasmata* (Archaea), *Phycisphaerae*, *Planctomycetacia*, *Nitrospira* and *Nitrospinia*. The presence of these microbial groups is associated with low oxygen concentrations, which can occur at intermittent depths ranging from approximately 130 to 1000 m in the water column (Bandekar et al., 2018; Durbin & Teske, 2012). Additionally, these taxa are linked to nitrogen cycle metabolism (Bandekar et al., 2018; Daims & Wagner, 2018; Sánchez et al., 2018; Spring et al., 2018; Wei & Yu, 2018; Zhao et al., 2019), except for *Thermoplasmata*. The samples grouped within cluster two (C15, G35, K55, O74 and O76) have depths greater than 100 m, and the D.O. levels ranged from 2.63 to 3.94 mg L^−1^; the depth and D.O. values (Table S1) recorded for these stations explain the presence of the aforementioned genera. Therefore, the results of the PERMANOVA analysis confirm the effect of depth (*R* ^2^ = 0.63, *p* = 0.011) and D.O. (*R* ^2^ = 0.056, *p* = 0.016) on shifts in bacterial community composition, indicating a relationship between depth and oxygen concentration values. This relationship aligns with previous reports in the nGoM, where water depth was correlated with changes in temperature, oxygen concentration, light penetration, hydrostatic pressure and microbial community composition (Schauer et al., 2003).

The PERMANOVA and one‐way ANOVA (Tables S3 and S4) analyses revealed that variations in *Chl‐a* concentration impact community changes. *Synechococcus* and *Prochlorococcus* have previously been identified as primary sources of *Chl‐a* in tropical and subtropical oligotrophic zones (Hewson, Capone, et al., 2006; Jasna et al., 2020; Medina et al., 2019; Pehr et al., 2018). *Chl‐a* concentrations ranged from 0.20 to 1.57 mg m^3^ in the water column of the Yucatan Shelf area (Table 2). As indicated by studies conducted by Medina et al. (2019, 2020), upwelling events can enhance planktonic groups that contribute to *Chl‐a* production, leading to a wide range of concentrations for this parameter.

In the SIMPER analysis, changes in relative abundances of *Synechococcus* and *Prochlorococcus* were associated with the grouping of clusters one, three and four. Additionally, the relative abundances of the genera NS4 marine group, NS5 marine group and NS2b marine group varied among clusters one, three and four (Figure 6). In contrast, these genera were scarcely detected in cluster two, where *Chl‐a* concentrations ranged from 0.3 to 0.6 mg L^−1^. These results are consistent with previous studies that have noted a positive relationship between these groups and *Chl‐a* production (Reyes et al., 2019; Seong et al., 2021).

The highest relative abundance of the NS4 marine group and NS5 marine group was observed in surface water from transect O with the exception of station O76 (Figure 5A). Conversely, in bottom water, the NS2b marine group show up the higher relative abundance in transect O (Figure 5B). Recent studies have noted a relationship between *Chl‐a* concentration and increases in the relative abundances of the aforementioned genera (Reyes et al., 2019; Seong et al., 2021). In transect O, *Chl‐a* concentrations ranged from 1.3 to 2 mg m^3^ (Table S1). Previous observations in this area have indicated the presence of Cape Catoche's upwelling, which can promote high *Chl‐a* levels (Perugini et al., 2007). Thus, we can infer that the presence of Cape Catoche's upwelling is associated with the highest abundances of the NS4 marine group, NS5 marine group and NS2b marine group in transect O. Furthermore, the Venn diagram revealed a higher number of unshared taxa in transect O (Figure 7; Table S5), which may also be attributed to the presence of Cape Catoche's upwelling, leading to an improvement in nutrient levels.

### Estimation of hydrocarbon degradation activity and detection of putative hydrocarbon‐degrading taxa

The European Union regulation stipulates that for ports and estuaries, the maximum allowable concentration of TPHs is 300 μg L^−1^ (Boehm & Flest, 1982). The obtained values during the GOMEX V oceanographic campaign show a maximum concentration of 0.72 μg L^−1^ for surface water and 0.86 μg L^−1^ for bottom water (Table 3). These values fall below the permissible levels in the water column, suggesting that hydrocarbons are not a pollution source in the YS area. In the sGoM, concentrations of 5 to 106,000 μg L^−1^ 3 years after the Ixtoc spill in 1979 were measured when evaluating the water column (Biller et al., 2015). Therefore, no impact of oil spills has been detected in the area. However, despite low hydrocarbon concentration, potential hydrocarbon‐degrading taxa were detected in both surface and bottom water, as well as increases in the number of copies of the *alkB* gene and increments in the abundances of CHB (Table 3). Hence, the presence of hydrocarbon‐degrading taxa in the YS area under low TPHs concentration can be associated with the potential of hydrocarbon‐degrading bacteria to degrade extra‐membrane compounds such as polysaccharides (Dombrowski et al., 2016; Love et al., 2013; Marine, 2017). Alternatively, the low input of crude oil from natural seeps and routine oil transport operations stimulates the growth of microorganisms specialised in oil degradation (Mapelli et al., 2017; Scoma et al., 2017; Won et al., 2017).

The genera *Alteromonas*, *Acinetobacter*, *Balneola*, *Erythrobacter*, *Oleibacter*, *Roseibacillus*, and the MWH‐UniP1 aquatic group showed relative abundances increases on the surface water of stations C12, C15, G31, G32, K51, K52, K53, K55, O73, O76 (Figure 5A). Previous studies have associated the presence of these genera in marine environments that have been anthropogenically impacted (Wang et al., 2016), especially with hydrocarbons (Baltar et al., 2018; Carney, 2009; Liu et al., 2017, 2019; Miller, 2019; Su et al., 2019; Valencia Agami et al., 2019; Wang et al., 2016; Xue et al., 2021). Thus, these results suggest punctual oil pollution in these sites during sampling. Likewise, in the stations mentioned above, increases in the number of copies of the *alkB* gene and the abundances of CHB (Table 3) were also observed, which in early works has been linked to degradation activity (Régimbeau et al., 2021; Uribe Flores et al., 2019). Moreover, these results are supported by the fluoranthene‐pyrene relationship, that confirmed the existence of petrogenic residues in the YS water column and sediments, according with the reports of Árcega‐Cabrera and Dótor‐Almazán (2021).

It is worth mentioning that the genera *Alteromonas*, *Acinetobacter*, *Balneola*, *Erythrobacter*, *Oleibacter*, *Roseibacillus*, and the MWH‐UniP1 aquatic group were observed in different combinations (Figure S2) for each of the mentioned stations (C12, C15, G31, G32, K51, K52, K53, K55, O73, O76). The various combinations observed at each station may be linked to the niche occupied by each taxon in the environment, allowing them to form a comprehensive metabolic network (Hazen et al., 2016; Su et al., 2019; Wang et al., 2016; Xue et al., 2021). The highest values of *alkB* and CHB (Table 3) were found at stations K53, K55, O74 and O76, coinciding with the elevated abundances of *Alteromonas* (Figure 5A and Figure S2). This pattern is particularly prominent at station O76, where *Alteromonas* represented 60% of the composition, and the highest values of *alkB* (126,189,389.35 copies mL^−1^) and CHB (24,000 CFU mL^−1^) were recorded. Previous mesocosm experiments using water from the sGoM have demonstrated the significant role of the genus *Alteromonas* in hydrocarbon degradation within this region (Régimbeau et al., 2021). While this outcome diverges from reports concerning the nGoM, where the most commonly reported genera for hydrocarbon degradation are *Alcanivorax*, *Cycloclasticus* and *Colwellia* (Hazen et al., 2016; Kimes et al., 2014; Linda et al., 2018), the genus *Alteromonas* appears to hold greater significance in the sGoM. The measurements obtained for *alkB* and CHB in bottom water (Table 3) suggest that the greatest degradation activity occurs in surface water. However, further experimental studies are needed to confirm this. These studies could involve experimental investigations using laboratory and mesocosm setups to simulate oil spills. Additionally, incorporating methods such as controlled release experiments, isotopic labelling techniques, and microbial community profiling could provide a more comprehensive understanding of the impact of oil spills on bacterioplankton communities.

## CONCLUSIONS

The results obtained in this study contribute to the understanding of the bacterioplankton community in the Yucatan Shelf of the sGoM. Physicochemical parameters, such as depth, dissolved oxygen, and chlorophyll‐a, were the variables explaining the changes in beta diversity. Furthermore, despite the low hydrocarbon concentration values detected in samples from the Yucatan Shelf water column, the results suggest a potential response of the bacterial community to small oil spill events, as indicated by enriched cultures of hydrocarbon‐degrading bacteria (CHB), the expression of the *alkB* gene detected by qPCR, and the presence of genera such as *Alteromonas*, *Acinetobacter*, *Balneola*, *Erythrobacter*, *Oleibacter*, *Roseibacillus* and the MWH‐UniP1 aquatic group. These genera have previously been recognised as potential hydrocarbon degraders. Additionally, changes in relative abundances and increases in *alkB* and CHB values suggest that *Alteromonas* could be considered the keystone for hydrocarbon degradation in the studied area.

## AUTHOR CONTRIBUTIONS

**Sonia S. Valencia‐Agami:** Conceptualization (equal); data curation (lead); formal analysis (lead); investigation (equal); methodology (lead); software (lead); validation (lead); visualization (lead); writing – original draft (lead); writing – review and editing (equal). **Daniel Cerqueda‐García:** Conceptualization (equal); data curation (equal); formal analysis (lead); investigation (equal); software (lead); validation (lead); visualization (equal); writing – review and editing (equal). **Abril M. Gamboa‐Muñoz:** Data curation (supporting); methodology (equal); validation (supporting); visualization (supporting); writing – review and editing (equal). **M. Leopoldina Aguirre‐Macedo:** Conceptualization (equal); funding acquisition (lead); investigation (equal); methodology (supporting); project administration (lead); resources (lead); supervision (lead); writing – original draft (supporting); writing – review and editing (lead). **José Q. García‐Maldonado:** Conceptualization (equal); funding acquisition (lead); investigation (equal); methodology (supporting); project administration (equal); resources (lead); supervision (lead); writing – original draft (supporting); writing – review and editing (lead).

## CONFLICT OF INTEREST STATEMENT

The authors declare no conflict of interest.

## Supporting information


**Figure S1:** DPCoA calculated with Unweighted Unifrac distances. Bottom samples are represented by circles and surface samples are represented by triangles. Colour in the figure represents the different sample transects.

**Figure S2:** Relative abundance of putative hydrocarbon‐degrading bacteria in surface water.

**Table S1:** Physicochemical variables from the surface (5 m depth) and bottom water samples (from 10 to 176 m depth).

**Table S2:** Linear regression summary for measure the effect of the TPH concentration over CHB and *alkB* gene.

**Table S3:** Summary of the one‐way ANOVA among the Clusters and Chlorophyll‐a concentrations.

**Table S4:** Tukey Test of the one‐way ANOVA among the Clusters and Chlorophyll‐a concentrations.

**Table S5:** Taxonomical assignment and abundances per transect (C, G, K, and O) of the ASVs identified from the Venn diagram.

## Data Availability

All sequences obtained from this study have been deposited in the National Center for Biotechnology Information (NCBI) Sequence Read Archive under accession number PRJNA895031.
