# Supplementary figures and images for: Structure and composition of microbial communities in the water column from Southern Gulf of Mexico and detection of putative hydrocarbon‐degrading microorganisms
Source: Environ Microbiol Rep. 2024 May 1;16(3):e13264. doi: 10.1111/1758-2229.13264 (PMC11062854; doi:10.1111/1758-2229.13264)

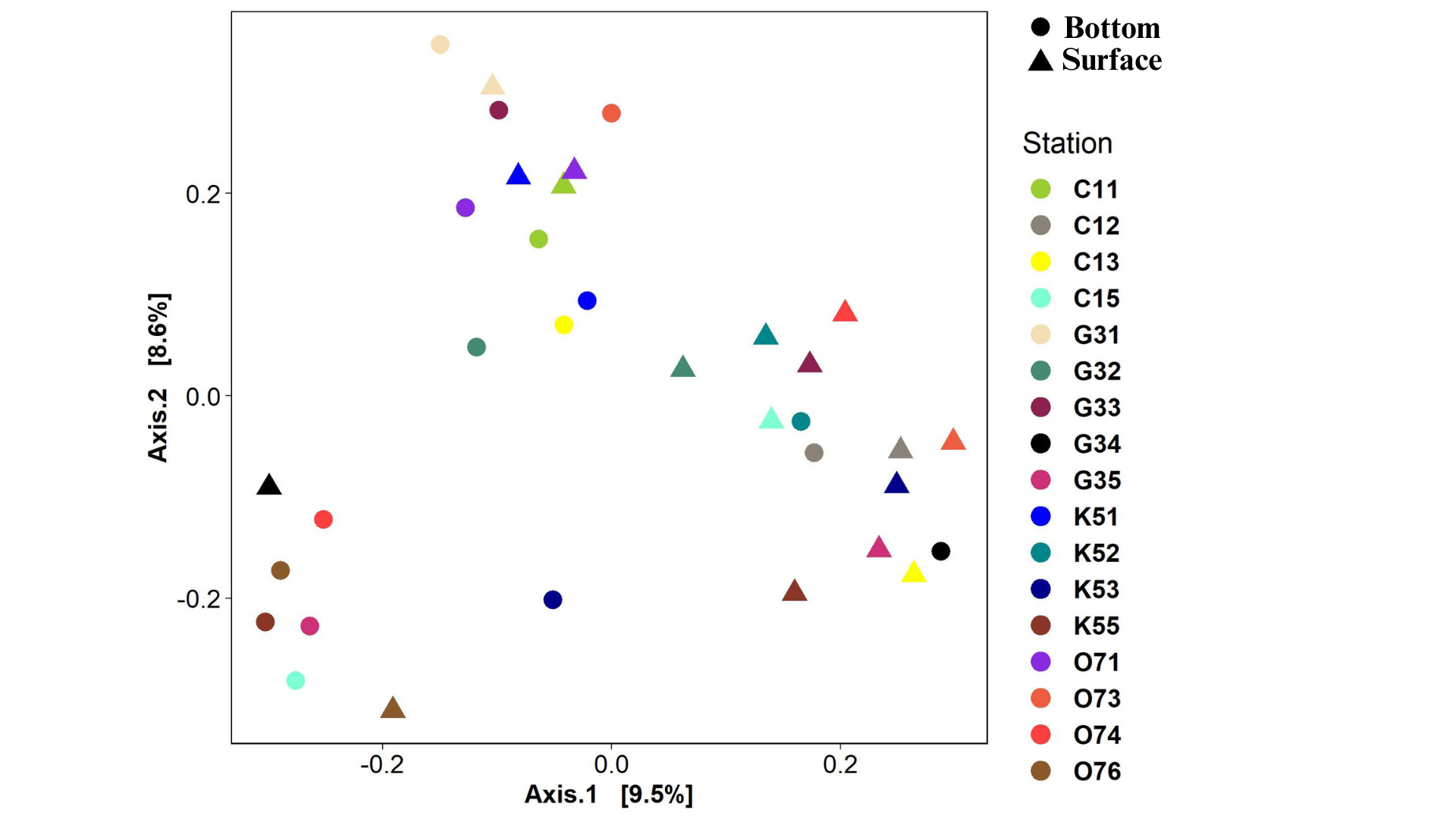

Supplement: Supplementary file 1 — Figure S1: DPCoA calculated with Unweighted Unifrac distances. Bottom samples are represented by circles and surface samples are represented by triangles. Colour in the figure represents the different sample transects. [file EMI4-16-e13264-s003.png]

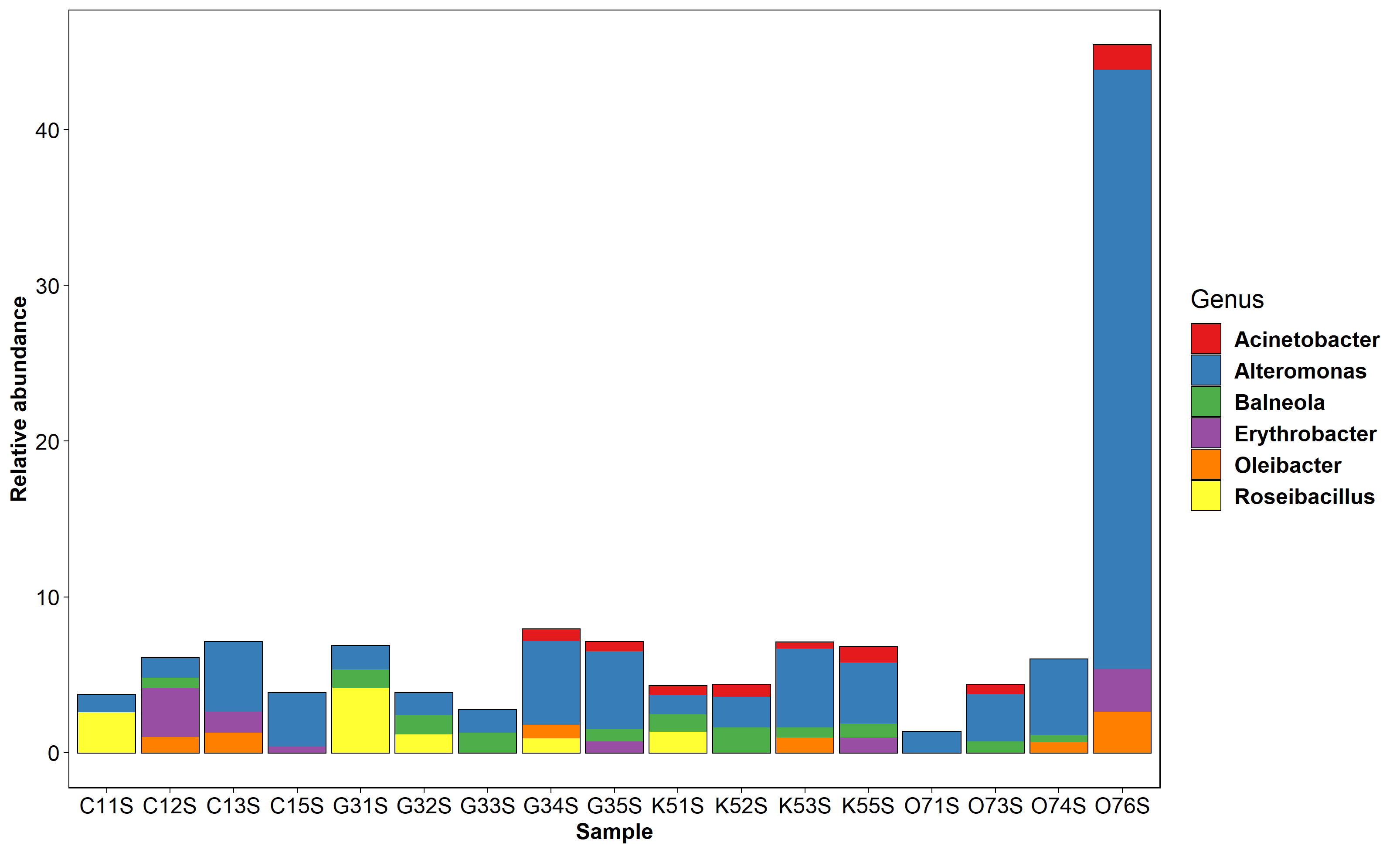

Supplement: Supplementary file 2 — Figure S2: Relative abundance of putative hydrocarbon‐degrading bacteria in surface water. [file EMI4-16-e13264-s002.tiff]
