# Supplementary material for: Structure and composition of microbial communities in the water column from Southern Gulf of Mexico and detection of putative hydrocarbon‐degrading microorganisms
Source: Environ Microbiol Rep. 2024 May 1;16(3):e13264. doi: 10.1111/1758-2229.13264 (PMC11062854; doi:10.1111/1758-2229.13264)
Supplement: Supplementary file 3 — Table S1: Physicochemical variables from the surface (5 m depth) and bottom water samples (from 10 to 176 m depth). [file EMI4-16-e13264-s001.docx]

**Table S1.**  Physicochemical variables from the surface (5 m depth) and bottom water samples (from 10 to 176 m depth). A '-' was used to indicate that this data was not measured during the oceanographic campaign. D.O = Dissolved Oxygen, Chl-a = Chlorophyll-a

| Station | Depth (m) | | Chl-a  (mg m^3^) | D.O  (mgL^-1^) | T°  (°C) | Salinity  (g Kg^-1^) | pH | NO^3^  (µmol L^-1^) | PO^4^  (µmol L^-1^) | SiO^4^  (µmol L^-1^) |
| --- | --- | --- | --- | --- | --- | --- | --- | --- | --- | --- |
| C11 | | 5 | 0.5 | 3.42 | 27.24 | 36.44 | - | 1.037 | 0.085 | 1.622 |
| C11 | | 16 | 0.6 | 2.89 | 26.18 | 36.45 | - | 1.015 | 0.088 | 1.345 |
| C12 | | 5 | 0.4 | 4.37 | 29.15 | 36.53 | 9.02 | 1.336 | 0.096 | 1.115 |
| C12 | | 37 | 0.52 | 4.67 | 26.74 | 36.43 | 9.14 | 1.087 | 0.060 | 1.842 |
| C13 | | 5 | 0.3 | 4.29 | 29.9 | 36.58 | 9.03 | 1.087 | 0.092 | 1.152 |
| C13 | | 49 | 1.57 | 4.47 | 23.89 | 36.45 | 9.07 | 0.759 | 0.057 | 1.354 |
| C15 | | 5 | 0.3 | 4.26 | 30.12 | 36.59 | - | 2.104 | 0.040 | 1.171 |
| C15 | | 150 | 0.3 | 2.58 | 16.65 | 36.2 | - | 6.795 | 0.032 | 1.235 |
| G31 | | 5 | 0.5 | 4.73 | 26.68 | 36.44 | - | 0.693 | 0.033 | 1.086 |
| G31 | | 10 | 0.8 | 4.57 | 24.12 | 36.45 | - | 0.451 | 0.104 | 1.207 |
| G32 | | 5 | 0.5 | 4.43 | 28.89 | 36.42 | 6.93 | 1.399 | 0.081 | 1.068 |
| G32 | | 27 | 1.05 | 4.79 | 24.42 | 36.4 | 6.85 | 0.633 | 0.063 | 1.960 |
| G33 | | 5 | 0.4 | 4.36 | 29.39 | 36.34 | - | 0.442 | 0.040 | 1.060 |
| G33 | | 44 | 1.57 | 3.92 | 22.6 | 36.51 | - | 0.521 | 0.096 | 1.400 |
| G34 | | 5 | 0.3 | 4.31 | 23.84 | 36.09 | 9.02 | 0.904 | 0.090 | 1.057 |
| G34 | | 85 | 0.58 | 3.73 | 21.94 | 36.5 | 9.11 | 2.582 | 0.506 | 1.071 |
| G35 | | 5 | 0.2 | 4.27 | 30.38 | 36.47 | - | 0.905 | 0.055 | 1.070 |
| G35 | | 143 | 0.3 | 2.64 | 17.94 | 36.39 | - | 13.090 | 0.269 | 6.470 |
| K51 | | 5 | 0.2 | 4.85 | 25.94 | 36.41 | - | 0.704 | 0.084 | 1.097 |
| K51 | | 16 | 0.6 | 4.16 | 23.85 | 36.5 | - | 0.673 | 0.087 | 1.945 |
| K52 | | 5 | 0.3 | 4.51 | 27.92 | 36.35 | 6.73 | 0.417 | 0.092 | 1.226 |
| K52 | | 32 | 1 | 4.53 | 24.31 | 36.43 | 6.73 | 0.713 | 0.205 | 1.961 |
| K53 | | 5 | 0.2 | 4.27 | 30.44 | 35.86 | - | 0.230 | 0.107 | 1.241 |
| K53 | | 45 | 1.57 | 4.15 | 23.43 | 36.52 | - | 1.122 | 0.303 | 1.035 |
| K55 | | 5 | 0.2 | 4.28 | 30 | 35.92 | - | 0.98 | 0.027 | 1.074 |
| K55 | | 103 | 0.52 | 3.17 | 19.53 | 36.48 | - | 2.781 | 0.094 | 2.487 |
| O71 | | 5 | 0.6 | 4.65 | 24.13 | 36.46 | - | 1.075 | 0.060 | 1.141 |
| O71 | | 17 | 1.3 | 4.37 | 23.18 | 36.49 | - | 1.030 | 0.049 | 1.268 |
| O73 | | 5 | 0.2 | 4.4 | 29.74 | 36.22 | 7 | 0.574 | 0.034 | 1.077 |
| O73 | | 24 | 2 | 5.07 | 26.1 | 36.46 | 7 | 0.805 | 0.321 | 1.347 |
| O74 | | 5 | 0.2 | 4.3 | 30.17 | 35.9 | 6.89 | 0.472 | 0.051 | 1.045 |
| O74 | | 65 | 1.3 | 4.16 | 22.07 | 36.48 | 6.56 | 2.93 | 0.033 | 1.054 |
| O76 | | 5 | 0.3 | 4.24 | 30.29 | 35.58 | 6.9 | 0.635 | 0.061 | 1.074 |
| O76 | | 179 | - | 3.18 | 16.99 | 36.48 | 6.8 | 2.198 | 0.739 | 1.050 |
