# Supplementary material for: Structure and composition of microbial communities in the water column from Southern Gulf of Mexico and detection of putative hydrocarbon‐degrading microorganisms
Source: Environ Microbiol Rep. 2024 May 1;16(3):e13264. doi: 10.1111/1758-2229.13264 (PMC11062854; doi:10.1111/1758-2229.13264)
Supplement: Supplementary file 4 — Table S2: Linear regression summary for measure the effect of the TPH concentration over CHB and alkB gene. [file EMI4-16-e13264-s004.docx]

**Table S2.** Linear regression summary for measure the effect of the TPH concentration over CHB and *alkB* gene. SE = Standard Error and R^2^= Coefficient of determination.

| X variable | Y  Variable | Depth | Intercept | SE | R^2^ | P value |
| --- | --- | --- | --- | --- | --- | --- |
| TPH | CHB | Surface | -18096.6 | 5719.5 | 0.05 | 0.18 |
| TPH | *alkB* gene | Surface | -19065.8 | 7899.9 | 0.03 | 0.10 |
| TPH | CHB | Bottom | -13607.3 | 3957.2 | 0.05 | 0.35 |
| TPH | *alkB* gene | Bottom | -17557.2 | 9384.3 | 0.10 | 0.25 |
