# Supplementary material for: Structure and composition of microbial communities in the water column from Southern Gulf of Mexico and detection of putative hydrocarbon‐degrading microorganisms
Source: Environ Microbiol Rep. 2024 May 1;16(3):e13264. doi: 10.1111/1758-2229.13264 (PMC11062854; doi:10.1111/1758-2229.13264)
Supplement: Supplementary file 5 — Table S3: Summary of the one‐way ANOVA among the Clusters and Chlorophyll‐a concentrations. [file EMI4-16-e13264-s007.docx]

**Table S3.** Summary of the one-way ANOVA among the Clusters and Chlorophyll-a concentrations. Using 95% confidence level (Df = Degrees of freedom, SS = Sums of squares, MS= Mean squares, and F value = F-statistic)

|  | Df | SS | MS | F value | Pr(>F) |
| --- | --- | --- | --- | --- | --- |
| *Chl-a* | 1 | 4.86 | 4.86 | 5.42 | 0.02 |
| Residuals | 32 | 28.66 | 0.89 |  |  |
