# Supplementary material for: Structure and composition of microbial communities in the water column from Southern Gulf of Mexico and detection of putative hydrocarbon‐degrading microorganisms
Source: Environ Microbiol Rep. 2024 May 1;16(3):e13264. doi: 10.1111/1758-2229.13264 (PMC11062854; doi:10.1111/1758-2229.13264)
Supplement: Supplementary file 6 — Table S4: Tukey Test of the one‐way ANOVA among the Clusters and Chlorophyll‐a concentrations. [file EMI4-16-e13264-s005.docx]

**Table S4.** Tukey Test of the one-way ANOVA among the Clusters and Chlorophyll-a concentrations. Using 95% confidence level (diff = differences between the means, lwr =lower bound of the confidence interval, upr = upper bound of the confidence interval, P adj= adjusted p-values for each pairwise comparison).

| Cluster  comparison | diff | lwr | upr | P adj |
| --- | --- | --- | --- | --- |
| 2-1 | -0.09 | -1.65 | 2.45 | 0.05 |
| 3-1 | -0.03 | -1.46 | 1.39 | 0.99 |
| 4-1 | 0.98 | -0.46 | 2.43 | 0.99 |
| 3-2 | 0.06 | -1.11 | 1.24 | 0.05 |
| 4-2 | 1.08 | -.0.11 | 2.28 | 0.05 |
| 4-3 | 1.02 | -0.01 | 2.05 | 0.09 |
