# Supplementary material for: Structure and composition of microbial communities in the water column from Southern Gulf of Mexico and detection of putative hydrocarbon‐degrading microorganisms
Source: Environ Microbiol Rep. 2024 May 1;16(3):e13264. doi: 10.1111/1758-2229.13264 (PMC11062854; doi:10.1111/1758-2229.13264)
Supplement: Supplementary file 7 — Table S5: Taxonomical assignment and abundances per transect (C, G, K, and O) of the ASVs identified from the Venn diagram. [file EMI4-16-e13264-s006.pdf]

Table S5. Taxonomical assignment and abundances per transect (C, G, K, and O) of the ASVs identified from the Venn diagram.

Row.names	Domain	Phylum	Class	Order	Family	Genus	Species	Taxonomy	Tax	C	G	K	O
01303439956	Archaea	Thaumarchae	Marine Group	Unknown Ord	Unknown Fam	Candidatus Ni	Unassigned	Marine Group	Marine Group	0	0.00606667	0	0.0048
03bcc070ee5	Bacteria	Proteobacteri	Alphaproteob	Rhodospirillal	Rhodospirillac	uncultured	Ambiguous_ta	Alphaproteob	Alphaproteob	0	0.00833333	0.0048	0.0032
045f1925adb	Bacteria	Proteobacteri	Alphaproteob	Rhodobactera	Rhodobactera	Unassigned	Unassigned	Alphaproteob	Alphaproteob	0	0.02493333	0.00753333	0.0244
05d628a2c22	Bacteria	Proteobacteri	Gammaprote	Gammaprote	Unknown Fam	uncultured	uncultured ba	Gammaprote	Gammaprote	0	0.0048	0	0.0028
06132633f45	Bacteria	Proteobacteri	Gammaprote	Oceanospirilla	SAR86 clade	uncultured ba	uncultured ba	Gammaprote	Gammaprote	0.009	0.0204	0.01026667	0
098a8dec261	Bacteria	Actinobacteria	Acidimicrobiia	Acidimicrobial	OM1 clade	Candidatus Ac	uncultured ba	Acidimicrobiia	Acidimicrobiia	0.0252	0.02193333	0.02093333	0
0c12008afe23	Bacteria	Proteobacteri	Alphaproteob	Rhodobactera	Rhodobactera	uncultured	uncultured ba	Alphaproteob	Alphaproteob	0.02646667	0	0.01746667	0.05186667
0d16ae4c875	Archaea	Euryarchaeota	Thermoplasm	Thermoplasm	Marine Group	uncultured ar	uncultured ar	Thermoplasm	Thermoplasm	0	0.00526667	0.00913333	0
0d27ff39dab4	Bacteria	Marinimicrobi	Unassigned	Unassigned	Unassigned	Unassigned	Unassigned	Unassigned U	Unassigned-U	0	0	0.0182	0.01753333
0ed93be8baf	Archaea	Euryarchaeota	Thermoplasm	Thermoplasm	Marine Group	Unassigned	Unassigned	Thermoplasm	Thermoplasm	0.00573333	0.00713333	0	0
0f90efa93b62	Bacteria	Proteobacteri	Alphaproteob	SAR11 clade	Unassigned	Unassigned	Unassigned	Alphaproteob	Alphaproteob	0	0	0	0.02393333
0fe1bf0bd654	Bacteria	Marinimicrobi	Unassigned	Unassigned	Unassigned	Unassigned	Unassigned	Unassigned U	Unassigned-U	0	0	0	0.02966667
104e96a39fb	Bacteria	Proteobacteri	Gammaprote	Alteromonada	Alteromonada	Alteromonas	Unassigned	Gammaprote	Gammaprote	0.04853333	0.07786667	0.12313333	0.22346667
109188847b9	Bacteria	Cyanobacteria	Cyanobacteria	SubsectionI	FamilyI	Synechococcu	Unassigned	Cyanobacteria	Cyanobacteria	0.06433333	0.02933333	0.0442	0
10d60fa0cf24	Bacteria	Proteobacteri	Alphaproteob	SAR11 clade	Surface 1	uncultured ba	uncultured ba	Alphaproteob	Alphaproteob	0	0.04793333	0.017	0.0364
10e0dfa3736f	Bacteria	Proteobacteri	Alphaproteob	Rickettsiales	SAR116 clade	Unassigned	Unassigned	Alphaproteob	Alphaproteob	0	0.00813333	0.0244	0.01873333
10f5d3c146e	Bacteria	Proteobacteri	Alphaproteob	Sphingomona	Erythrobacter	Erythrobacter	Unassigned	Alphaproteob	Alphaproteob	0.01746667	0	0	0
11ae5934143	Bacteria	Actinobacteria	Acidimicrobiia	Acidimicrobial	OM1 clade	Candidatus Ac	uncultured ba	Acidimicrobiia	Acidimicrobiia	0.05866667	0.0638	0.06746667	0.015
1421f1c3a1a2	Bacteria	Cyanobacteria	Cyanobacteria	SubsectionI	FamilyI	Prochlorococc	Unassigned	Cyanobacteria	Cyanobacteria	0.06766667	0.0256	0.07746667	0.04353333
16b5bd2c80f	Bacteria	Proteobacteri	Alphaproteob	SAR11 clade	Surface 1	uncultured ba	uncultured ba	Alphaproteob	Alphaproteob	0.04953333	0.06946667	0.128	0.02746667
1977c130422	Bacteria	Proteobacteri	Alphaproteob	SAR11 clade	Surface 1	Unassigned	Unassigned	Alphaproteob	Alphaproteob	0.0358	0.01993333	0.11286667	0.1192
1ae51740df0	Bacteria	Proteobacteri	Deltaproteoba	SAR324 clade(	uncultured ba	uncultured ba	uncultured ba	Deltaproteoba	Deltaproteoba	0.00473333	0	0	0.00193333
1b67d2fb001	Bacteria	Proteobacteri	Gammaprote	Oceanospirilla	SAR86 clade	uncultured ba	uncultured ba	Gammaprote	Gammaprote	0.00453333	0.00513333	0.00773333	0.01066667
1b9cff406b7d	Bacteria	Bacteroidetes	Flavobacteriia	Flavobacterial	Flavobacteria	NS2b marine	Ambiguous_ta	Flavobacteriia	Flavobacteriia	0.0104	0.02733333	0	0.04486667
1d5c36dccf88	Bacteria	Bacteroidetes	Flavobacteriia	Flavobacterial	Flavobacteria	NS5 marine gr	Unassigned	Flavobacteriia	Flavobacteriia	0	0.00733333	0.01193333	0.0218
20b29a337a7	Bacteria	Actinobacteria	Acidimicrobiia	Acidimicrobial	Sva0996 mari	uncultured ba	uncultured ba	Acidimicrobiia	Acidimicrobiia	0.0164	0.01486667	0.00673333	0
20ecb64a096	Archaea	Euryarchaeota	Thermoplasm	Thermoplasm	Marine Group	Unassigned	Unassigned	Thermoplasm	Thermoplasm	0	0.00473333	0.003	0
21217b93342	Bacteria	Proteobacteri	Gammaprote	Oceanospirilla	SAR86 clade	Unassigned	Unassigned	Gammaprote	Gammaprote	0.0052	0	0	0.01046667
224c9cedc1cf	Bacteria	Proteobacteri	AEGEAN-245	Unassigned	Unassigned	Unassigned	Unassigned	AEGEAN-245 U	AEGEAN-245-	0	0	0.00473333	0.009
2286272d050	Bacteria	Cyanobacteria	Cyanobacteria	SubsectionI	FamilyI	Prochlorococc	Unassigned	Cyanobacteria	Cyanobacteria	0.107	0.0406	0.13713333	0.05353333
22acb0db3f2	Bacteria	Bacteroidetes	Flavobacteriia	Flavobacterial	Flavobacteria	NS5 marine gr	Unassigned	Flavobacteriia	Flavobacteriia	0.01793333	0	0.03986667	0
2457199b8f7	Bacteria	Proteobacteri	Alphaproteob	Rhodobactera	Rhodobactera	Unassigned	Unassigned	Alphaproteob	Alphaproteob	0	0.0066	0	0.0436
24a9eef22c0f	Bacteria	Cyanobacteria	Cyanobacteria	SubsectionI	FamilyI	Synechococcu	Unassigned	Cyanobacteria	Cyanobacteria	0.15306667	0.20873333	0.04333333	0.07306667
25a57650972	Bacteria	Verrucomicro	Arctic97B-4 m	Unassigned	Unassigned	Unassigned	Unassigned	Arctic97B-4 m	Arctic97B-4 m	0.00333333	0.0068	0	0
27ffa636f31a	Bacteria	Proteobacteri	Alphaproteob	Rhodospirillal	Rhodospirillac	AEGEAN-169	Unassigned	Alphaproteob	Alphaproteob	0.05466667	0.01346667	0.0566	0.025
282d9a68c0e	Bacteria	Marinimicrobi	Unassigned	Unassigned	Unassigned	Unassigned	Unassigned	Unassigned U	Unassigned-U	0.00646667	0.0096	0	0
29a7c65de41	Bacteria	Proteobacteri	Gammaprote	Gammaprote	Unknown Fam	uncultured	Unassigned	Gammaprote	Gammaprote	0	0	0.00433333	0.01026667
2ab4ce9736d	Bacteria	Cyanobacteria	Cyanobacteria	SubsectionI	FamilyI	Synechococcu	Unassigned	Cyanobacteria	Cyanobacteria	0.01386667	0.0206	0	0
2c240285631	Bacteria	Actinobacteria	Acidimicrobiia	Acidimicrobial	OM1 clade	Candidatus Ac	uncultured ba	Acidimicrobiia	Acidimicrobiia	0.07093333	0.07806667	0.10333333	0.02666667
2d164c11db6	Bacteria	Bacteroidetes	Flavobacteriia	Flavobacterial	Flavobacteria	NS4 marine gr	Unassigned	Flavobacteriia	Flavobacteriia	0.02153333	0.02326667	0.01553333	0
2d6aeb3edfa	Bacteria	Proteobacteri	Alphaproteob	Rhodobactera	Rhodobactera	uncultured	uncultured ba	Alphaproteob	Alphaproteob	0.022	0	0.0148	0.0148
2e2845b88ee	Bacteria	Proteobacteri	Gammaprote	Oceanospirilla	SAR86 clade	uncultured ba	uncultured ba	Gammaprote	Gammaprote	0	0.00866667	0	0.0096
2f8c9053118	Bacteria	Cyanobacteria	Chloroplast	Unassigned	Unassigned	Unassigned	Unassigned	Chloroplast U	Chloroplast-U	0.00333333	0.00146667	0	0
300de36a72a	Bacteria	Proteobacteri	Alphaproteob	Rhizobiales	Rhodobiaceae	Rhodobium	Unassigned	Alphaproteob	Alphaproteob	0	0.00406667	0.00193333	0.04973333
30859c9e393	Bacteria	Proteobacteri	Gammaprote	Oceanospirilla	SAR86 clade	uncultured ba	uncultured ba	Gammaprote	Gammaprote	0.01866667	0.02453333	0.02046667	0.00913333
30e296e85d4	Bacteria	Cyanobacteria	Cyanobacteria	SubsectionI	FamilyI	Synechococcu	Unassigned	Cyanobacteria	Cyanobacteria	0.04426667	0.06546667	0.0244	0
32bdc1a8d1a	Bacteria	Proteobacteri	Alphaproteob	SAR11 clade	Surface 1	Unassigned	Unassigned	Alphaproteob	Alphaproteob	0.0338	0.04086667	0	0.0324
338f0b0f2e3c	Bacteria	Proteobacteri	Gammaprote	Oceanospirilla	SAR86 clade	Unassigned	Unassigned	Gammaprote	Gammaprote	0	0.01046667	0.00646667	0.0054
33f25c0a68c5	Bacteria	Proteobacteri	Gammaprote	E01-9C-26 ma	Unassigned	Unassigned	Unassigned	Gammaprote	Gammaprote	0	0	0	0.0066
3535dd8f4bf0	Bacteria	Proteobacteri	Alphaproteob	Rickettsiales	SAR116 clade	Unassigned	Unassigned	Alphaproteob	Alphaproteob	0.00906667	0.00813333	0	0
3541ee5e624	Archaea	Euryarchaeota	Thermoplasm	Thermoplasm	Marine Group	uncultured ma	uncultured ma	Thermoplasm	Thermoplasm	0	0.00533333	0	0.00226667
36b9abde0b7	Bacteria	Proteobacteri	Deltaproteoba	SAR324 clade(	Unassigned	Unassigned	Unassigned	Deltaproteoba	Deltaproteoba	0	0.00413333	0	0.00606667
38641c88d60	Bacteria	Proteobacteri	Gammaprote	Alteromonada	Alteromonada	Alteromonas	Unassigned	Gammaprote	Gammaprote	0.11273333	0.0644	0.0836	0.3296
3b5bd56a5e4	Bacteria	Proteobacteri	Gammaprote	Oceanospirilla	SAR86 clade	uncultured ba	uncultured ba	Gammaprote	Gammaprote	0.01566667	0.0098	0.01133333	0.03
3d57758f2de	Bacteria	Actinobacteria	Acidimicrobiia	Acidimicrobial	OM1 clade	Candidatus Ac	Unassigned	Acidimicrobiia	Acidimicrobiia	0.03213333	0.0336	0.02606667	0
3da31ce69bd	Bacteria	Proteobacteri	Alphaproteob	Rhodobactera	Rhodobactera	uncultured	Unassigned	Alphaproteob	Alphaproteob	0.0542	0.02886667	0.02	0
3ff3c5958f42	Bacteria	Proteobacteri	Gammaprote	Thiotrichales	Thiotrichacea	Thiothrix	Unassigned	Gammaprote	Gammaprote	0.00573333	0.0066	0.00926667	0
4085ea691a1	Bacteria	Proteobacteri	Gammaprote	Oceanospirilla	SAR86 clade	uncultured ba	uncultured ba	Gammaprote	Gammaprote	0	0	0.0422	0.0178
43421b387b4	Bacteria	Proteobacteri	Gammaprote	Salinisphaeral	Salinisphaera	ZD0417 marin	Unassigned	Gammaprote	Gammaprote	0.0058	0	0.00973333	0.01566667
43624370f66	Bacteria	Verrucomicro	Verrucomicro	Verrucomicro	DEV007	Unassigned	Unassigned	Verrucomicro	Verrucomicro	0.00266667	0.0102	0	0
47f7bf8b8db6	Bacteria	Planctomycet	Pla3 lineage	Unassigned	Unassigned	Unassigned	Unassigned	Pla3 lineage U	Pla3 lineage-U	0.00366667	0.0018	0	0
493d16eb620	Bacteria	Proteobacteri	Gammaprote	Oceanospirilla	SAR86 clade	Unassigned	Unassigned	Gammaprote	Gammaprote	0	0	0	0.00666667
4a3ea3e317a	Bacteria	Cyanobacteria	Cyanobacteria	SubsectionI	FamilyI	Prochlorococc	Unassigned	Cyanobacteria	Cyanobacteria	0	0	0	0.07413333
4b4179ffe62a	Bacteria	Proteobacteri	Alphaproteob	SAR11 clade	Surface 2	Unassigned	Unassigned	Alphaproteob	Alphaproteob	0.06413333	0.0834	0.10753333	0.02893333
4ddfbb0cb3e	Bacteria	Actinobacteria	Acidimicrobiia	Acidimicrobial	OM1 clade	Candidatus Ac	uncultured ba	Acidimicrobiia	Acidimicrobiia	0.0318	0.01346667	0.01026667	0
4e4357e589e	Bacteria	Marinimicrobi	Unassigned	Unassigned	Unassigned	Unassigned	Unassigned	Unassigned U	Unassigned-U	0.01546667	0.0714	0.053	0.04226667
4f86e3d9318	Bacteria	Proteobacteri	Alphaproteob	SAR11 clade	Unassigned	Unassigned	Unassigned	Alphaproteob	Alphaproteob	0.0246	0.0462	0.06033333	0.05253333
4fd44fa32d0a	Bacteria	Bacteroidetes	Flavobacteriia	Flavobacterial	Flavobacteria	NS5 marine gr	Unassigned	Flavobacteriia	Flavobacteriia	0	0.0032	0	0.00493333
502b57bc909	Bacteria	Bacteroidetes	Flavobacteriia	Flavobacterial	Flavobacteria	NS4 marine gr	Unassigned	Flavobacteriia	Flavobacteriia	0	0.05166667	0	0
503fc4e5f1fe1	Bacteria	Proteobacteri	Gammaprote	Oceanospirilla	SAR86 clade	Unassigned	Unassigned	Gammaprote	Gammaprote	0.04593333	0.0622	0	0
507f4a8cb6d	Bacteria	Proteobacteri	Gammaprote	Oceanospirilla	SAR86 clade	uncultured ba	uncultured ba	Gammaprote	Gammaprote	0.003	0	0	0.0016
50c035f42497	Bacteria	Verrucomicro	Verrucomicro	Verrucomicro	DEV007	Unassigned	Unassigned	Verrucomicro	Verrucomicro	0.03026667	0.01246667	0	0
514ee6e3657	Bacteria	Proteobacteri	Alphaproteob	Rhodospirillal	Rhodospirillac	AEGEAN-169	Unassigned	Alphaproteob	Alphaproteob	0.02526667	0.04093333	0.03793333	0.03
51b5d785fbe	Bacteria	Proteobacteri	Gammaprote	Oceanospirilla	JL-ETNP-Y6	Unassigned	Unassigned	Gammaprote	Gammaprote	0.02193333	0.02773333	0.03373333	0
54ad0f5ba93	Bacteria	Proteobacteri	Alphaproteob	Rhodospirillal	Rhodospirillac	AEGEAN-169	Unassigned	Alphaproteob	Alphaproteob	0.0198	0.02873333	0.0384	0.06313333
5566a384111	Bacteria	Cyanobacteria	Cyanobacteria	SubsectionI	FamilyI	Synechococcu	Unassigned	Cyanobacteria	Cyanobacteria	0	0	0.02313333	0.06533333
5c8caae697e	Bacteria	Proteobacteri	Gammaprote	Cellvibrionales	Halieaceae	OM60(NOR5)	Unassigned	Gammaprote	Gammaprote	0.0116	0.03386667	0	0
5d60320b38c	Bacteria	Proteobacteri	Alphaproteob	SAR11 clade	Surface 1	uncultured ba	uncultured ba	Alphaproteob	Alphaproteob	0.00906667	0.019	0.0808	0.03066667
600e6d7544b	Bacteria	Proteobacteri	Alphaproteob	Rhodobactera	Rhodobactera	Unassigned	Unassigned	Alphaproteob	Alphaproteob	0.00253333	0.00293333	0	0
6448f24d39b	Bacteria	Cyanobacteria	Cyanobacteria	SubsectionI	FamilyI	Prochlorococc	Unassigned	Cyanobacteria	Cyanobacteria	0.02846667	0.03766667	0.082	0.0378
6496cb96f51	Bacteria	Cyanobacteria	Cyanobacteria	SubsectionI	FamilyI	Synechococcu	Unassigned	Cyanobacteria	Cyanobacteria	0.01526667	0.0182	0.01333333	0.0094
64ff49f5b156	Bacteria	Cyanobacteria	Cyanobacteria	SubsectionI	FamilyI	Synechococcu	Unassigned	Cyanobacteria	Cyanobacteria	0.23326667	0.36346667	0.126	0.1166
69d1a138b0f	Bacteria	Proteobacteri	Alphaproteob	Rickettsiales	SAR116 clade	Unassigned	Unassigned	Alphaproteob	Alphaproteob	0.0058	0.00746667	0.0164	0
6a79ed45c60	Bacteria	Actinobacteria	Acidimicrobiia	Acidimicrobial	Acidimicrobia	Unassigned	Unassigned	Acidimicrobiia	Acidimicrobiia	0.0042	0.0068	0	0
6c333f1c8048	Bacteria	Proteobacteri	Gammaprote	Oceanospirilla	SAR86 clade	Unassigned	Unassigned	Gammaprote	Gammaprote	0.0072	0.02406667	0.0352	0.01866667
6e01a05adc0	Bacteria	Proteobacteri	Gammaprote	Thiotrichales	Thiotrichacea	Thiothrix	Unassigned	Gammaprote	Gammaprote	0.01	0	0.0112	0.01066667
6e6ad52ba41	Bacteria	Proteobacteri	Gammaprote	Cellvibrionales	Porticoccacea	SAR92 clade	Unassigned	Gammaprote	Gammaprote	0	0	0	0.06526667
72282666b7d	Bacteria	Proteobacteri	Gammaprote	Oceanospirilla	SAR86 clade	Unassigned	Unassigned	Gammaprote	Gammaprote	0.06213333	0.09133333	0.08333333	0.075
72822261b03	Bacteria	Actinobacteria	Acidimicrobiia	Acidimicrobial	Sva0996 mari	Unassigned	Unassigned	Acidimicrobiia	Acidimicrobiia	0	0.0414	0.02706667	0
7293014787d	Archaea	Euryarchaeota	Thermoplasm	Thermoplasm	Marine Group	uncultured ar	uncultured ar	Thermoplasm	Thermoplasm	0.0074	0.00986667	0.0058	0
7596249444d	Archaea	Euryarchaeota	Thermoplasm	Thermoplasm	Marine Group	uncultured ar	uncultured ar	Thermoplasm	Thermoplasm	0.00206667	0	0	0
780606d5c90	Bacteria	Bacteroidetes	Flavobacteriia	Flavobacterial	Flavobacteria	NS4 marine gr	uncultured ba	Flavobacteriia	Flavobacteriia	0.0124	0.00546667	0	0.01406667

78bd4f149d25	Bacteria		Bacteroidetes	Cytophagia	Cytophagales	Flammeovirga	Marinoscillum	Unassigned	Cytophagia M	Cytophagia-M	0.00526667	0	0	0
7adb2646f964	Bacteria		Proteobacteri	Gammaprote	Salinisphaeral	Salinisphaerac	ZD0417 marin	Unassigned	Gammaprote	Gammaprote	0	0.019	0.0084	0.01026667
7d161d99ed4	Bacteria		Verrucomicro	Verrucomicrol	Verrucomicrol	DEV007	Unassigned	Unassigned	Verrucomicrol	Verrucomicrol	0.00946667	0.0056	0	0
80b3674372f0	Bacteria		Proteobacteri	Betaproteoba	Burkholderial	Alcaligenacea	MWH-UniP1 a	uncultured ba	Betaproteoba	Betaproteoba	0.00773333	0.00306667	0	0
82660f6f9681	Bacteria		Proteobacteri	Alphaproteob	SAR11 clade	Surface 1	uncultured ba	uncultured ba	Alphaproteob	Alphaproteob	0	0.027	0.05053333	0.03813333
83a2017d310	Bacteria		Proteobacteri	Gammaprote	Cellvibrionales	Halieaceae	OM60(NOR5)	Unassigned	Gammaprote	Gammaprote	0.01526667	0.01533333	0.0128	0
8b981225fb90	Bacteria		Proteobacteri	Alphaproteob	SAR11 clade	Surface 1	Unassigned	Unassigned	Alphaproteob	Alphaproteob	0.02253333	0.01606667	0.0306	0.0414
8c23e1b11f3f	Bacteria		Proteobacteri	Gammaprote	Alteromonada	Alteromonada	Alteromonas	Unassigned	Gammaprote	Gammaprote	0.01513333	0.05833333	0.06413333	0.13313333
8dac47b4863a	Bacteria		Proteobacteri	Alphaproteob	Rhodospirillal	Rhodospirillac	AEGEAN-169 r	uncultured ba	Alphaproteob	Alphaproteob	0.07433333	0.06686667	0.02253333	0.00846667
8e3a0b6d616	Bacteria		Cyanobacteria	Cyanobacteria	SubsectionI	FamilyI	Synechococcu	Unassigned	Cyanobacteria	Cyanobacteria	0.2862	0.30913333	0.10686667	0.1152
8f0cfc797e96	Bacteria		Proteobacteri	Alphaproteob	SAR11 clade	Surface 2	Unassigned	Unassigned	Alphaproteob	Alphaproteob	0.06126667	0.06513333	0.0604	0.02673333
9235b00318a	Bacteria		Proteobacteri	Alphaproteob	Rhizobiales	Rhodobiaceae	Rhodobium	Unassigned	Alphaproteob	Alphaproteob	0	0	0.01506667	0.04346667
92d1276ce57	Bacteria		Bacteroidetes	Flavobacteriia	Flavobacterial	Flavobacteria	NS4 marine gr	Unassigned	Flavobacteriia	Flavobacteriia	0.04793333	0.01346667	0	0
92e48924fea0	Bacteria		Proteobacteri	Alphaproteob	SAR11 clade	Surface 1	Unassigned	Unassigned	Alphaproteob	Alphaproteob	0.11206667	0.12066667	0.16233333	0.1226
96b673b22dd	Bacteria		Bacteroidetes	Flavobacteriia	Flavobacterial	Flavobacteria	NS2b marine g	Unassigned	Flavobacteriia	Flavobacteriia	0.0032	0.01866667	0	0
96df805bf7a8	Bacteria		Proteobacteri	Alphaproteob	Caulobacteral	Hyphomonada	uncultured	Unassigned	Alphaproteob	Alphaproteob	0	0.009	0.01186667	0
979c8cafa8d5	Bacteria		Proteobacteri	Alphaproteob	Rhodospirillal	Rhodospirillac	AEGEAN-169 r	Unassigned	Alphaproteob	Alphaproteob	0.06626667	0.0804	0.07573333	0.06666667
9978c5bed10	Bacteria		Proteobacteri	Gammaprote	Oceanospirilla	SAR86 clade	uncultured ba	uncultured ba	Gammaprote	Gammaprote	0.01606667	0.0596	0	0.00466667
9b4965ca6e2	Bacteria		Proteobacteri	Alphaproteob	SAR11 clade	Surface 1	uncultured ba	uncultured ba	Alphaproteob	Alphaproteob	0.0154	0.01233333	0.01626667	0.005
9c9adf7c10a9	Bacteria		Bacteroidetes	Flavobacteriia	Flavobacterial	Flavobacteria	NS2b marine g	Unassigned	Flavobacteriia	Flavobacteriia	0	0.009	0	0.0076
9cc398b3486f	Bacteria		Proteobacteri	Alphaproteob	SAR11 clade	Unassigned	Unassigned	Unassigned	Alphaproteob	Alphaproteob	0.0312	0.00646667	0.04146667	0
9db42a8129d	Bacteria		Proteobacteri	Alphaproteob	Rhizobiales	PS1 clade	Unassigned	Unassigned	Alphaproteob	Alphaproteob	0	0.01453333	0.02906667	0.01766667
9ee3f78db262	Bacteria		Proteobacteri	Alphaproteob	Rhodobactera	Rhodobactera	uncultured	Unassigned	Alphaproteob	Alphaproteob	0.03173333	0	0.03086667	0.02773333
9f8bd0af9effa	Bacteria		Proteobacteri	Alphaproteob	Rhodospirillal	Rhodospirillac	AEGEAN-169 r	Unassigned	Alphaproteob	Alphaproteob	0.01	0	0.03953333	0.00806667
a236563a64e	Archaea		Thaumarchae	Marine Group	Unknown Ord	Unknown Fam	Unassigned	Unassigned	Marine Group	Marine Group	0	0.0042	0.00246667	0
a2eef912ceb0	Bacteria		Proteobacteri	Gammaprote	Alteromonada	Alteromonada	Alteromonas	Unassigned	Gammaprote	Gammaprote	0.03233333	0.04066667	0.0226	0.135
a4bbd054d76	Bacteria		Proteobacteri	Gammaprote	Oceanospirilla	SAR86 clade	uncultured ba	uncultured ba	Gammaprote	Gammaprote	0.02013333	0.0222	0.03573333	0.01986667
a4f83a3f2ad6	Bacteria		Proteobacteri	Alphaproteob	Rhodospirillal	Rhodospirillac	AEGEAN-169 r	uncultured ba	Alphaproteob	Alphaproteob	0.06693333	0.07013333	0.06033333	0.04646667
a5ab5d32888	Bacteria		Bacteroidetes	Bacteroidetes	Order III	Unknown Fam	Balneola	Unassigned	Bacteroidetes	Bacteroidetes	0	0.00766667	0.00233333	0.0028
a661f13e240a	Bacteria		Proteobacteri	Gammaprote	Oceanospirilla	SAR86 clade	Unassigned	Unassigned	Gammaprote	Gammaprote	0	0.01346667	0	0.0414
a73101e5be5	Bacteria		Proteobacteri	Gammaprote	E01-9C-26 ma	Unassigned	Unassigned	Unassigned	Gammaprote	Gammaprote	0.01693333	0.0348	0	0
a73530b5817	Bacteria		Bacteroidetes	Flavobacteriia	Flavobacterial	Flavobacteria	Unassigned	Unassigned	Flavobacteriia	Flavobacteriia	0	0.00466667	0.01886667	0
a761c8b3a93	Bacteria		Proteobacteri	Gammaprote	Alteromonada	Alteromonada	Alteromonas	Unassigned	Gammaprote	Gammaprote	0	0	0.00606667	0.02306667
a81e12babef	Bacteria		Proteobacteri	Alphaproteob	Rhodobactera	Rhodobactera	uncultured	Unassigned	Alphaproteob	Alphaproteob	0	0.00686667	0.02106667	0.0266
a8b57fcefaaa	Bacteria		Cyanobacteria	Cyanobacteria	SubsectionI	FamilyI	Synechococcu	Unassigned	Cyanobacteria	Cyanobacteria	0.12486667	0.22533333	0.0416	0.07233333
a9aa4e828e5	Bacteria		Bacteroidetes	Flavobacteriia	Flavobacterial	Flavobacteria	NS4 marine gr	Unassigned	Flavobacteriia	Flavobacteriia	0.0074	0.0306	0.01033333	0.01266667
aaead0c48b4	Bacteria		Proteobacteri	Alphaproteob	SAR11 clade	Deep 1	uncultured ba	uncultured ba	Alphaproteob	Alphaproteob	0.03226667	0.02046667	0	0
ac9fc7a89185	Bacteria		Actinobacteria	Acidimicrobiia	Acidimicrobial	Sva0996 mari	Unassigned	Unassigned	Acidimicrobiia	Acidimicrobiia	0	0.00513333	0	0.00913333
b0214253b0d	Bacteria		Proteobacteri	Alphaproteob	SAR11 clade	Surface 1	Unassigned	Unassigned	Alphaproteob	Alphaproteob	0	0.015	0.01366667	0.02326667
b06861341f64	Bacteria		Proteobacteri	Alphaproteob	Rhodobactera	Rhodobactera	Unassigned	Unassigned	Alphaproteob	Alphaproteob	0	0.01086667	0	0.006
b4ca07204bc3	Bacteria		Proteobacteri	Alphaproteob	Rhodospirillal	Rhodospirillac	AEGEAN-169 r	Unassigned	Alphaproteob	Alphaproteob	0.01233333	0.01213333	0.05153333	0.01653333
b4d497cb5bd	Bacteria		Nitrospinae	Nitrospinia	Nitrospinales	Nitrospinacea	uncultured	uncultured ba	Nitrospinia un	Nitrospinia-ur	0.0242	0	0	0.00193333
b4f1d1033db	Bacteria		Proteobacteri	Gammaprote	Oceanospirilla	SAR86 clade	Unassigned	Unassigned	Gammaprote	Gammaprote	0.05186667	0.03393333	0.0596	0.0294
b535f83882d7	Bacteria		Actinobacteria	Acidimicrobiia	Acidimicrobial	Unassigned	Unassigned	Unassigned	Acidimicrobiia	Acidimicrobiia	0.00573333	0.014	0	0
b5e2232142b	Bacteria		Proteobacteri	Alphaproteob	SAR11 clade	Unassigned	Unassigned	Unassigned	Alphaproteob	Alphaproteob	0.04326667	0.0442	0.0432	0.07333333
b726f276bafd	Bacteria		Proteobacteri	Alphaproteob	SAR11 clade	Unassigned	Unassigned	Unassigned	Alphaproteob	Alphaproteob	0	0.00793333	0.006	0.0026
bbf36805d9af	Bacteria		Proteobacteri	Alphaproteob	SAR11 clade	Surface 1	Unassigned	Unassigned	Alphaproteob	Alphaproteob	0.11573333	0.17246667	0.15766667	0.1018
bdc4cf49d3f8	Bacteria		Proteobacteri	Alphaproteob	SAR11 clade	Surface 1	Unassigned	Unassigned	Alphaproteob	Alphaproteob	0.01366667	0.00566667	0.0168	0.0104
bfeac2088a8b	Bacteria		Cyanobacteria	Cyanobacteria	SubsectionI	FamilyI	Prochlorococc	Unassigned	Cyanobacteria	Cyanobacteria	0	0	0	0.04493333
c027665bb35	Bacteria		Proteobacteri	Gammaprote	Oceanospirilla	SAR86 clade	uncultured ba	uncultured ba	Gammaprote	Gammaprote	0.00373333	0.00426667	0	0.00693333
c07fbab16872	Bacteria		Proteobacteri	Gammaprote	Oceanospirilla	SAR86 clade	Unassigned	Unassigned	Gammaprote	Gammaprote	0.00633333	0.0114	0.00906667	0.00573333
c085fa8077a9	Bacteria		Bacteroidetes	Flavobacteriia	Flavobacterial	Flavobacteria	NS4 marine gr	uncultured ba	Flavobacteriia	Flavobacteriia	0.01246667	0.01413333	0	0.0166
c11a13c54047	Bacteria		Proteobacteri	Alphaproteob	Rhodobactera	Rhodobactera	uncultured	uncultured ba	Alphaproteob	Alphaproteob	0.0228	0.01153333	0.0164	0
c5f54ce11999	Bacteria		Proteobacteri	Deltaproteoba	NB1-j	Unassigned	Unassigned	Unassigned	Deltaproteoba	Deltaproteoba	0.00486667	0	0	0.00353333
ca5e290ed88	Bacteria		Proteobacteri	Gammaprote	Oceanospirilla	JL-ETNP-Y6	Unassigned	Unassigned	Gammaprote	Gammaprote	0.0168	0.01833333	0	0.00906667
cd9c712ddcc6	Bacteria		Proteobacteri	Gammaprote	Oceanospirilla	SAR86 clade	Unassigned	Unassigned	Gammaprote	Gammaprote	0.00846667	0.00713333	0.0154	0.0164
cf5afe0bc995	Bacteria		Cyanobacteria	Cyanobacteria	SubsectionI	FamilyI	Synechococcu	Unassigned	Cyanobacteria	Cyanobacteria	0.14493333	0.32506667	0.0628	0.09686667
cff9bb2d7104	Bacteria		Actinobacteria	Acidimicrobiia	Acidimicrobial	OM1 clade	Candidatus Ac	uncultured ba	Acidimicrobiia	Acidimicrobiia	0.05733333	0.08033333	0.08086667	0.03093333
d1218789548	Bacteria		Proteobacteri	Gammaprote	Cellvibrionales	Porticoccacea	SAR92 clade	Unassigned	Gammaprote	Gammaprote	0.0072	0.01586667	0.01006667	0.00786667
d128ecb064a	Archaea		Thaumarchae	Marine Group	Unknown Ord	Unknown Fam	Candidatus Ni	Unassigned	Marine Group	Marine Group	0	0.0078	0.012	0.00373333
d2c4e9ef319c	Bacteria		Proteobacteri	Gammaprote	Alteromonada	Alteromonada	Alteromonas	Unassigned	Gammaprote	Gammaprote	0.02366667	0.02346667	0.02566667	0.10853333
d3cb5f14951a	Bacteria		Proteobacteri	Gammaprote	Oceanospirilla	SAR86 clade	Unassigned	Unassigned	Gammaprote	Gammaprote	0.06033333	0.04653333	0.08606667	0.05273333
d6a07cab9c73	Bacteria		Proteobacteri	Alphaproteob	SAR11 clade	Deep 1	Unassigned	Unassigned	Alphaproteob	Alphaproteob	0	0.01606667	0	0.03906667
d779600b1e2	Bacteria		Proteobacteri	Gammaprote	Alteromonada	Pseudoalteror	Pseudoalteror	Unassigned	Gammaprote	Gammaprote	0	0	0.0096	0.00973333
d85422ab97b	Bacteria		Proteobacteri	Alphaproteob	SAR11 clade	Unassigned	Unassigned	Unassigned	Alphaproteob	Alphaproteob	0.01993333	0.01006667	0.0452	0.02426667
d8cc1fbd5a01	Bacteria		Proteobacteri	Alphaproteob	SAR11 clade	Surface 1	Unassigned	Unassigned	Alphaproteob	Alphaproteob	0.01866667	0.01093333	0.01966667	0.02566667
d91e66b17d8	Bacteria		Proteobacteri	Gammaprote	Oceanospirilla	SAR86 clade	uncultured ba	uncultured ba	Gammaprote	Gammaprote	0.0356	0.03453333	0.04333333	0.01786667
da42f301e96a	Bacteria		Proteobacteri	Gammaprote	Oceanospirilla	SAR86 clade	uncultured ba	uncultured ba	Gammaprote	Gammaprote	0.0984	0.0218	0.0418	0.00853333
dac166aabe0a	Bacteria		Proteobacteri	Alphaproteob	SAR11 clade	Surface 4	uncultured ba	uncultured ba	Alphaproteob	Alphaproteob	0.01666667	0.03813333	0.03286667	0.0072
dd5442f60a0b	Bacteria		Proteobacteri	Alphaproteob	SAR11 clade	Surface 1	uncultured ba	uncultured ba	Alphaproteob	Alphaproteob	0.09506667	0.05406667	0.0798	0.0556
dd626bdce4a	Bacteria		Bacteroidetes	Flavobacteriia	Flavobacterial	Flavobacteria	NS2b marine g	Unassigned	Flavobacteriia	Flavobacteriia	0.00813333	0.00493333	0.01553333	0
ddd62a567d2	Bacteria		Proteobacteri	Alphaproteob	SAR11 clade	Surface 1	uncultured ba	uncultured ba	Alphaproteob	Alphaproteob	0.00206667	0.0136	0.0078	0
df28112c4116	Bacteria		Proteobacteri	Gammaprote	Alteromonada	Alteromonada	Alteromonas	Unassigned	Gammaprote	Gammaprote	0	0.00513333	0.0048	0.0232
e00fa8978700	Archaea		Euryarchaeota	Thermoplasm	Thermoplasm	Marine Group	uncultured ar	uncultured ar	Thermoplasm	Thermoplasm	0	0.0076	0	0.01386667
e2815bb49ea	Bacteria		Proteobacteri	Alphaproteob	Rhodospirillal	Rhodospirillac	AEGEAN-169 r	uncultured ba	Alphaproteob	Alphaproteob	0	0.0062	0	0.0226
e62181e5438	Bacteria		Proteobacteri	Alphaproteob	Rhizobiales	PS1 clade	Unassigned	Unassigned	Alphaproteob	Alphaproteob	0	0.011	0.0138	0.06966667
e726bdafd34b	Archaea		Euryarchaeota	Thermoplasm	Thermoplasm	Marine Group	Unassigned	Unassigned	Thermoplasm	Thermoplasm	0.00986667	0.0336	0.00833333	0
ea59e6471a5	Bacteria		Cyanobacteria	Chloroplast	Unassigned	Unassigned	Unassigned	Unassigned	Chloroplast U	Chloroplast-U	0.00173333	0.0168	0	0
ec0bd6cbc5e4	Bacteria		Proteobacteri	Alphaproteob	Rhizobiales	Rhodobiaceae	Rhodobium	Unassigned	Alphaproteob	Alphaproteob	0	0.0114	0.00293333	0.01533333
ec39fa4bacac	Bacteria		Cyanobacteria	Cyanobacteria	SubsectionI	FamilyI	Synechococcu	Unassigned	Cyanobacteria	Cyanobacteria	0.1664	0.25713333	0.06326667	0.08493333
ed414c32ae8	Bacteria		Cyanobacteria	Cyanobacteria	SubsectionI	FamilyI	Prochlorococc	Unassigned	Cyanobacteria	Cyanobacteria	0.02873333	0.01373333	0.05153333	0.04953333
ed93cd70c2b0	Bacteria		Proteobacteri	Alphaproteob	Rickettsiales	S25-593	Unassigned	Unassigned	Alphaproteob	Alphaproteob	0	0	0.00766667	0.00926667
edd75adc4dc	Bacteria		Cyanobacteria	Cyanobacteria	SubsectionI	FamilyI	Synechococcu	Unassigned	Cyanobacteria	Cyanobacteria	0.01526667	0.02466667	0	0
ede00e7ef876	Bacteria		Proteobacteri	Gammaprote	Oceanospirilla	Oceanospirilla	Oleibacter	Unassigned	Gammaprote	Gammaprote	0.01373333	0	0.0068	0.0068
f324403d2242	Bacteria		Bacteroidetes	Flavobacteriia	Flavobacterial	Flavobacteria	NS5 marine gr	Unassigned	Flavobacteriia	Flavobacteriia	0.01706667	0.03053333	0	0
f54fe82f912f4	Bacteria		Proteobacteri	Gammaprote	Oceanospirilla	SAR86 clade	Unassigned	Unassigned	Gammaprote	Gammaprote	0.01286667	0.01146667	0	0
f629f149009b	Bacteria		Proteobacteri	Gammaprote	Oceanospirilla	SAR86 clade	uncultured ba	uncultured ba	Gammaprote	Gammaprote	0.02513333	0.04853333	0	0
f677bcc3ddef	Bacteria		Bacteroidetes	Sphingobacte	Sphingobacte	Saprospiracea	uncultured	Unassigned	Sphingobacte	Sphingobacte	7.33E-04	0	0.0028	0
f8d3a7079dac	Bacteria		Proteobacteri	Alphaproteob	Rhodospirillal	Rhodospirillac	uncultured	Unassigned	Alphaproteob	Alphaproteob	0	0.00213333	0.00293333	0
facdc48582c9	Bacteria		Proteobacteri	Alphaproteob	Rhodospirillal	Rhodospirillac	AEGEAN-169 r	Unassigned	Alphaproteob	Alphaproteob	0.03633333	0.0808	0.07433333	0.0518
fb9e491c54a3	Bacteria		Proteobacteri	Gammaprote	Oceanospirilla	SAR86 clade	Unassigned	Unassigned	Gammaprote	Gammaprote	0.03786667	0.0858	0.03966667	0.02013333
fd769187dc4e	Bacteria		Bacteroidetes	Flavobacteriia	Flavobacterial	Flavobacteria	NS4 marine gr	Unassigned	Flavobacteriia	Flavobacteriia	0	0.01606667	0	0.03873333
fdf4d66b6643	Bacteria		Cyanobacteria	Cyanobacteria	SubsectionI	FamilyI	Unassigned	Unassigned	Cyanobacteria	Cyanobacteria	0.01326667	0.0228	0.01513333	0

ff0d202b1dbe	Bacteria		Actinobacteri	Acidimicrobiia	Acidimicrobial	OM1 clade	Candidatus Ac	uncultured ba	Acidimicrobiia	Acidimicrobiia	0.02586667	0.02026667	0.02666667	0
ff212099b123	Bacteria		Proteobacteri	Alphaproteob	SAR11 clade	Surface 1	uncultured ba	uncultured ba	Alphaproteob	Alphaproteob	0.10806667	0.08946667	0.13006667	0.06893333
